# How do Modes of Data Collection Affect the Measurement of Demographic Key Indicators? Findings from a Three-Country Experiment Using the Generations and Gender Survey

**DOI:** 10.1007/s10680-026-09777-7

**Published:** 2026-08-13

**Authors:** Detlev Lück, Almut Schumann, Tom Emery, Peter Lugtig, Martin Bujard, Robert Naderi, Vera Toepoel, Susana Cabaço

**Affiliations:** 1 Federal Institute for Population Research (BiB), 65185 Wiesbaden, Germany; 2 Wiesbaden, Germany; 3 ODISSEI, T19-05, c/o Erasmus Universiteit Rotterdam, 3000 DR Rotterdam, Netherlands; 4 Methdology & Statistics, Utrecht University, 3584 CH Utrecht, Netherlands; 5 Institute of Medical Psychology, Medical Faculty, University Heidelberg, Heidelberg, Germany; 6 Statistics Netherlands (CBS), 6412 EX Heerlen, Netherlands; 7 FMO, 2593 HW Den Haag, Netherlands

**Keywords:** Survey mode, Face-to-face interviews, Computer assisted web interviews (CAWI), Measurement effect, Selection effect

## Abstract

Rising survey costs, increasing internet usage, and restrictions during the pandemic have accelerated a shift from face-to-face (Computer Assisted Personal Interviews, or CAPI) to web-based interviews (Computer Assisted Web Interviews, or CAWI). This paper analyses the consequences of this shift for the comparability of survey-based measurements of demographic key indicators. We use data from a unique experiment on the modes of data collection such as CAPI or CAWI (in the following: “modes”), conducted in the Generations and Gender Survey in Germany, Croatia, and Portugal. We calculate mode-specific means and ratios of selected indicators and perform regression analyses to estimate mode effects. In all countries, we find a few distinct mode-selection effects: Less educated people are more underrepresented in CAWI, and urban regions are underrepresented in CAPI mode. Mode-measurement effects vary widely across indicators and country contexts. For sensitive questions, there are less social desirability biases in CAWI. For questions with a high respondent burden, there are less survey satisficing biases in CAPI. Loop questions with a high respondent burden are of particular concern. The mode shift challenges comparability between data collections, in longitudinal analyses and in cross-country comparisons. Data users should use weights for descriptive analyses, control for the mode where possible, and consider mode differences when interpreting results. The unique data source allows insights into mode effects among demographic indicators that were not possible before.

**Supplementary Information:**

The online version contains supplementary material available at 10.1007/s10680-026-09777-7.

## Introduction

Research on family demography relies heavily on survey data. The accuracy of findings in demographic research is highly dependent on data quality, in particular the validity, reliability, and comparability of the available survey data. The question of how data quality is affected when survey designs change fundamentally is crucial. Such a change is currently underway. For a long time, the in-person face-to-face interview (computer-assisted personal interview, CAPI) has been considered the “gold standard” for scientific survey research and has been the dominant mode of data collection (Couper, 2011). This is particularly the case for panel studies, for which a primary objective has been to minimize attrition. In recent years, however, an increasing number of surveys has fully or partially adopted web-based modes of data collection (computer-assisted web interview, CAWI). This is true, for example, for “The General Social Survey (GSS)”, which transitioned from CAPI to CAWI in 2021 (Davern et al., 2021:10), as well as for the “UK Household Longitudinal Study (UKHLS)”, which introduced “a small amount of web interviewing” in 2015/2016 (Carpenter, 2017:1) and increased this share over the following three waves (Carpenter, 2017, 2018, 2019, 2020). We use the terms “mode” or “survey mode” to refer to the way of collecting information from respondents in a survey e.g. in-person face-to-face or as self-administered online interviews.

A particularly pertinent example for the field of demographic research is the Generations and Gender Survey (GGS) (Gauthier et al., 2025; Vikat et al., 2007). The GGS is an international panel survey on family demography that permits the investigation of issues pertaining to gender and generations over time and between countries. The GGS organized a first round of data collection (“GGS-I”) in 21 countries between 2004 and 2011, which was carried out in face-to-face mode. In 2020, the GGS started a new round of data collection (GGS-II) with fresh samples and an updated questionnaire (Gauthier et al., 2018, 2025). Of the 28 countries participating in this round by January 2026, nine are conducting the GGS in CAWI mode only, 10 are using a mixed-mode design that typically involves a mix CAWI and CAPI, while four are using CAPI exclusively (Generations & Gender Programme, 2024). As a consequence, a significant number of European countries that previously relied on face-to-face interviews as their primary source of empirical data on demography are now starting to rely on data collected online.

Despite the fact that there are good reasons for surveys to go online, the change of modes bears risks because the mode of data collection affects measurements. It does so in different ways in different studies (Cernat & Revilla, 2021; West et al., 2024). Analyses of trends over time may show jumps, while cross-national comparisons may indicate country differences that are both mere artefacts based on differences in the survey modes. Therefore, demographic research needs to know whether a change of mode has an effect on the measurement of its key indicators (see Hank & Kreyenfeld, 2016) and, if it does, which indicators and which correlations are affected in what way. These are the research questions addressed in this paper.

The Generations and Gender Programme (GGP), which is responsible for implementing the GGS, carried out a pilot study to assess mode effects in 2018 (Emery et al., 2026): In Germany, Croatia, and Portugal, an experiment was carried out, comparing a reference group that was interviewed face-to-face with an experimental group in which a “web-first” design was applied. Based on these unique data, we evaluate whether measurements of demographic key indicators differ significantly between the face-to-face mode and the “web-first” design. We compare univariate statistics to assess mode effects in general, binary logistic, and OLS regression analyses to assess mode-measurement effects as well as interaction effects of the mode with gender or country of data collection.

## Background

### The Generations and Gender Survey and its Shift towards Web Mode

The GGS is one of the largest internationally comparative panel studies on family demography. The topics covered by the GGS questionnaire include partnership history, fertility, children, separation and divorce, division of care work and household chores, external support with childcare and household chores, intergenerational ties and mutual support, and attitudes on family-related issues (Gauthier et al., 2025). The design of the GGS is a longitudinal panel design with three-year intervals between waves; the recommended number of panel waves is three (Gauthier et al., 2025). Interviews in GGS-I as well as in the 2018 pilot study lasted approximately one hour, strongly depending on the respondent’s biography. The target population in GGS-I was the residential population, aged 18 to 79. The mode of data collection was predominantly face-to-face (Gauthier et al., 2018).

Given that the data of GGS-I had become outdated, a restart with fresh samples was initiated for 2020 (GGS-II). For this restart, the questionnaire was significantly shortened and fundamentally revised in its content and structure. Loops were reduced as they had proved to be tiring for respondents with multiple children as well as for the interviewers. Technical improvements were implemented, specifically a pre-harmonized centrally programmed questionnaire, hosted on a server in the Netherlands for all data collections, thereby aiming to avoid the necessity for post-harmonization on questionnaire data. In countries where a survey of older populations, such as SHARE, was already in place, the age range was permitted to be reduced (Gauthier et al., 2025; Generations & Gender Programme, 2020).

A further innovation in GGS-II is the option to combine CAPI mode with CAWI, as a mixed-mode design, or to conduct the GGS fully online. This option was introduced at an early stage of the planning process, given that the costs associated with face-to-face interviews had become very high while response rates were declining over all survey modes (Luiten et al., 2020; Williams & Brick, 2018; Wolf et al., 2021). A GGP pilot study in Slovenia in 2011 (Petrič et al., 2011, 2012) prompted the GGP to consider this option, despite the length of the interview exceeding the recommended duration for CAWI interviews (Revilla & Höhne, 2020). To assess the design innovations of the GGS and its feasibility in CAWI mode, the GGP carried out a further pilot study in 2018 in Germany, Croatia, and Portugal (Emery et al., 2026). This paper is based on the data from this 2018 pilot study.

### Theories and Prior Research on Mode Effects

**Fig. 1:**
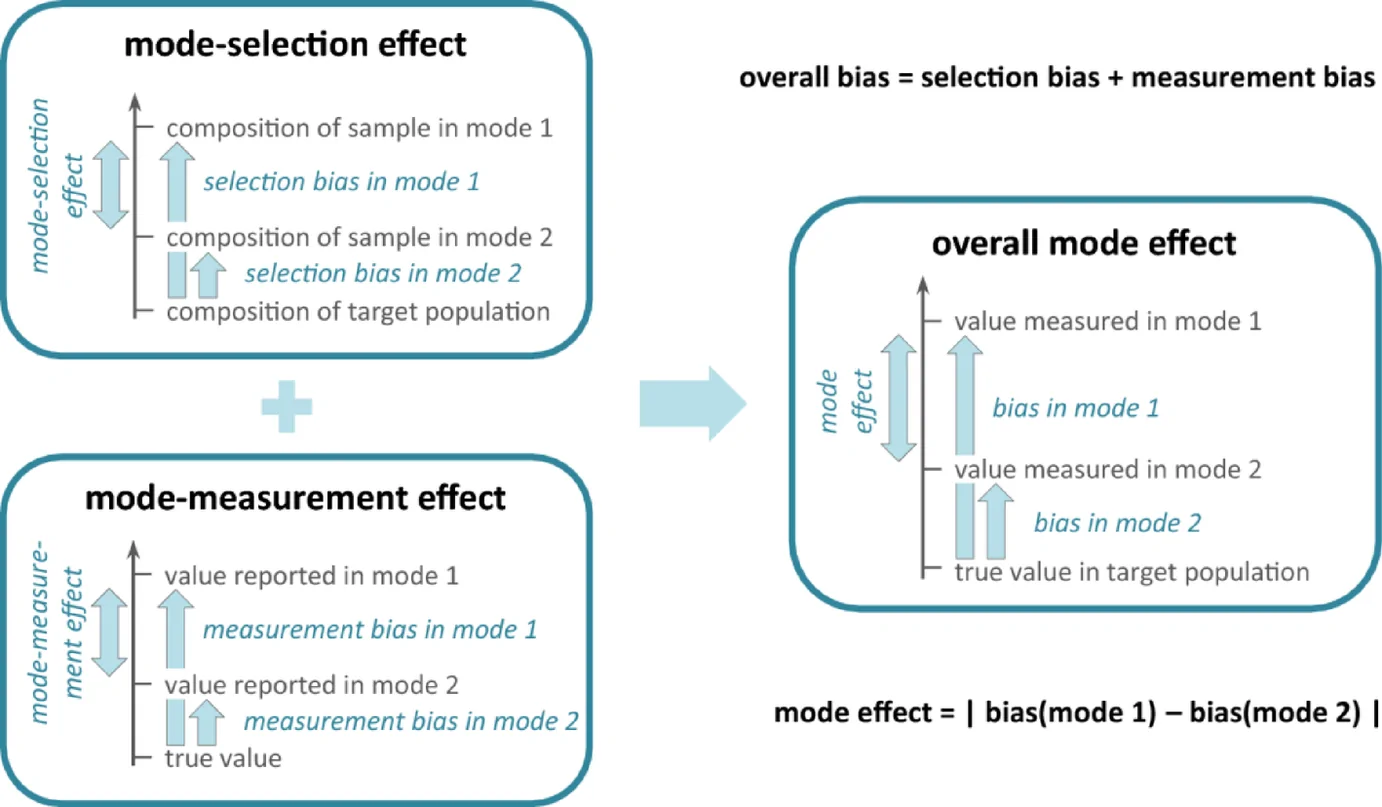
Mode-selection and mode-measurement effects. Note: *Own illustration*

The survey mode can impact data quality (e.g. Bianchi et al., 2017; Daikeler et al., 2020; Sakshaug et al., 2010). We refer to a difference in the measured outcome of two data collections (e.g. a mean or a ratio) conducted using two survey modes as “mode effect” (cp. Figure 1). The term must be distinguished from the mode-specific “bias”, which is the difference between the measured outcome of one data collection and the – usually unknown – true value in the target population. The mode effect equals the difference between the mode-specific biases of the two data collections, conducted in different modes (cp. Figure 1). Taken on its own, it does not reveal which of the two biases is bigger. Such mode effects occur for a number of reasons, which can be grouped into “media-related factors, factors influencing the information transmission, and interviewer effects” (Leeuw, 2005:244).

Mode effects are often distinguished into mode-selection and mode-measurement effects (see Vannieuwenhuyze et al., 2010). The *mode-selection effect* determines which respondents are more likely to participate in a particular survey mode. Usually, specific social groups are more likely to participate when a particular mode is offered than others, potentially resulting in biased distributions of variables that are mode-specific. The *mode-measurement effect* refers to the impact of the survey mode on the manner in which respondents provide answers during an interview (Vannieuwenhuyze et al., 2010). For various reasons, respondents may either refuse to answer a particular question (item nonresponse) or respond differently, depending on the mode they are interviewed in. The sum of mode-selection effect and mode-measurement effect equals the overall mode effect (see Fig. 1). Much more so than selection effects, measurement effects are undesirable because they lead to changes in outcomes that depend *only* on the interview mode (Heerwegh, 2009; Schumann & Lück, 2023). However, they are largely unavoidable, especially if face-to-face and self-administered modes are combined within a single survey (Cernat & Sakshaug, 2020; Leeuw, 2005). Accordingly, our research primarily focusses on measurement effects.

We highlight three mechanisms of measurement effects that we consider particularly relevant to our analyses, starting with two important interviewer effects: An interviewer may explain difficult questions and motivate the respondent to think of the correct answers, so that item nonresponse and misreporting are reduced in CAPI mode compared to CAWI mode (Galesic & Bosnjak, 2009; Hope et al., 2022). This effect can be explained by the theoretical concept of *satisficing* (Krosnick, 1991), which argues that respondents generally try to reduce the cognitive effort required to answer questions as far as they can without being identified as misreporting.

The presence of an interviewer may also increase the perceived pressure to give a socially desired answer to a question, thus increasing social desirability biases (Holbrook & Krosnick, 2010; Hope et al., 2022). Surveys often try to reduce social desirability by switching to “CASI mode” (computer-assisted self-interview) for sensitive questions within face-to-face interviewers: The interviewer gives the computer to the respondent who then fills in the answers on their own (Couper, 2005).

The third mechanism concerns an influence of auditory versus visual delivery of survey questions: If a respondent is not motivated enough to carefully consider all available answer categories, but rather applies satisficing strategies, CAPI and CAWI modes may promote two different types of response biases (Bogner & Landrock, 2016). A respondent listening to an interviewer reading out the response categories (in CAPI mode) may not remember the first answer categories well and therefore, when making a “quick decision”, choose one of the last categories mentioned, leading to a “recency effect”. When reading the answer options in the CAWI mode, an impatient respondent may in contrast choose one of the first categories, without taking the time to read to the end, resulting in a “primacy effect” (Bogner & Landrock, 2016).

### Theoretical Considerations and Expectations

Given that mode effects are question-specific and may be caused by different mechanisms happening simultaneously (Leeuw, 2005), we will not follow a strategy of testing hypotheses, but rather pursue a more explorative approach, assessing a large variety of demographic indicators and interpreting the findings ex post. Nevertheless, we have some a priori expectations towards the mode effects we should find (cp. also Table 1).

**Table 1:** Expectations towards mode differences

Indicator	Expected mode differences		
	Social desirability	Satisficing	Primacy/ recency
Having a partner	—	—	—
Having a same-sex relationship ^1^	web > f2f	—	—
Cohabiting	—	—	—
Being married	—	—	—
Number of biological children	—	f2f > web	—
Childless (no biological children)	—	web > f2f	—
Total number of children intended	f2f > web	—	—
Number of household members	—	f2f > web	—
Number of social network members	—	f2f > web	—
No social network member at all	—	web > f2f	—
Number of previous partners	—	f2f > web	—
No previous partner at all	—	web > f2f	—
Unprotected sex last 12 months ^2^	(f2f > web)	—	—
Unprot. sex last 12 months – item-nonresponse ^2^	(f2f > web)	web > f2f	—
Last sexual intercourse within last 4 weeks ^2^	(f2f > web)	—	—
Last time resp. had sex – item-nonresponse ^2^	(f2f > web)	web > f2f	—
Subjective infertility ^2^	(web > f2f)	—	f2f > web
Subjective infertility – item-nonresponse ^2^	(f2f > web)	web > f2f	—
Contraceptive use ^2^	(f2f > web)	—	web > f2f
Men doing equal share of laundry	f2f > web	—	—
Men doing equal share of preparing meals	f2f > web	—	—
Life satisfaction (11-point scale)	f2f > web	—	—
Happiness (11-point scale)	f2f > web	—	—

“f2f” = face-to-face interview (CAPI); “web” = online interview (CAWI).

(1) The indicator same-sex relationship will only be included in descriptive analyses because case numbers are too low for including it in the regression analyses.

(2) Question is answered by respondents themselves – instead of the interviewer – within the face-to-face interview to reduce pressure in terms of social desirability.

Effects in (parentheses) may not occur.

For indicators that are susceptible to social desirability biases, we expect the bias to be stronger in CAPI than in CAWI, in line with findings on relationship quality, relationship conflicts, and subjective well-being in the GGS (Piccitto et al., 2022; Schumann & Lück, 2023). This may be exemplified by the question on subjective infertility or the question regarding the last time the respondent had sexual intercourse. Aside from mode differences in the provided answers, we expect the ratios of item nonresponse to be higher in CAPI than in CAWI. For these questions (and for a few others), the interviewers had asked the respondents to fill out the questionnaire themselves in order to mitigate social desirability biases. It is possible, however, that mode differences may still be visible as the interviewer was present and may still have increased the subjectively perceived social pressure. We furthermore expect that mode effects due to social desirability will manifest in the proportion of reported same-sex relationships as well as in the questions on the intended total number of children, on the division of housework, on life satisfaction and on happiness.

For questions with a high respondent burden, we anticipate that biases resulting from satisficing will emerge, and we expect those biases to be more pronounced in CAWI than in CAPI. This may be the case for the reported number of biological children, with lower averages in CAWI, given that these are counted based on loops. Such loop designs were identified as a potential cause of under-reporting of children in the first round of GGS data collections (GGS-I), at least in Germany (Ruckdeschel et al., 2016). Furthermore, the start of loops has been found to bear a higher risk for interview break-offs (Emery et al., 2022). For the same reason, the reported number of previous partners and of household members may be lower in CAWI. We also expect that the reported size of a personal social network will be prone to satisficing. In this question, respondents were required to enter the names of each network member, which entailed greater effort and may have additionally prompted concerns regarding data protection. Furthermore, the question wording was lengthy (107 words).

Due to the auditory versus visual delivery of items, we expect to find recency effects in CAPI mode as well as primacy effects in CAWI among indicators with multiple answer categories. This may result in a lower reporting of contraceptive use in CAPI, given that the answer category indicating that no contraceptive method was used was the last one in a list of 15 answer categories. Likewise, it is possible that subjective infertility may be overreported in CAPI since the scale of four answer categories ranged from disagreement to agreement.

For the “web-first” design, we expect that measurements will be analogous to those obtained in CAWI mode, given that the majority of respondents (approximately 93%) in this experimental group have filled out the questionnaire online. Regarding country differences, we expect the findings to vary due to the demographic variations across these countries, in addition to the presence of slightly disparate selection biases. However, we do not expect any country-specific differences regarding the mode-measurement effects.

## Data and Methods

### The GGP Pilot Study and its Experiment on Modes

**Fig. 2:**
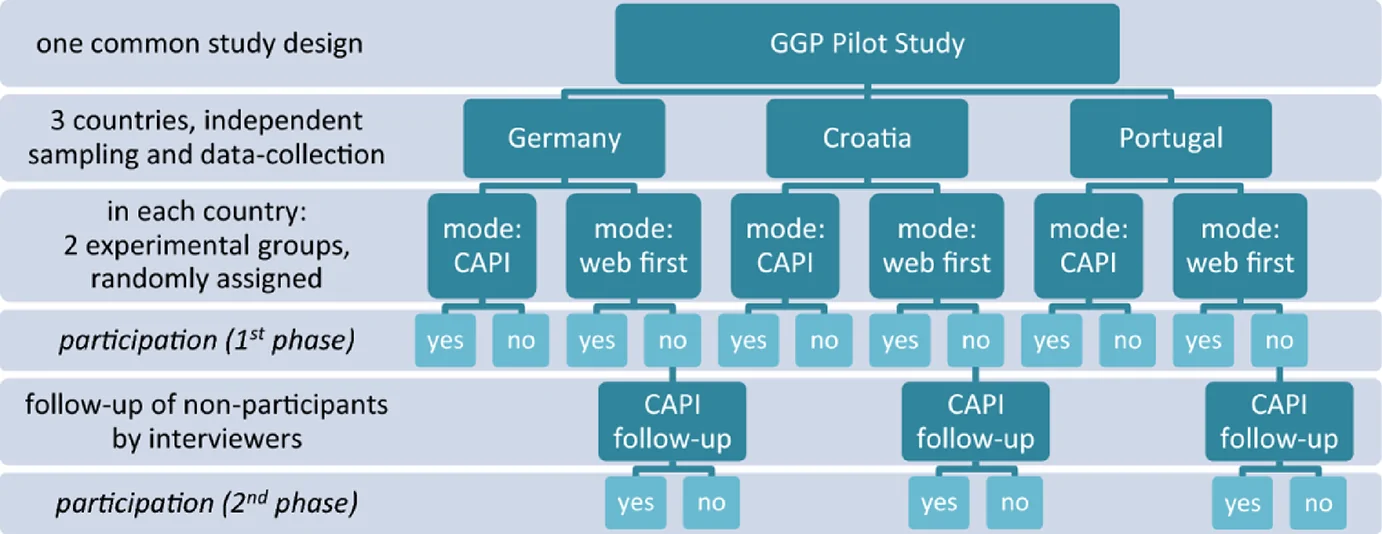
Experimental design. Note: *Own illustration. “CAPI” = “computer-assisted personal interviewing”, also referred to as “face-to-face interviewing”. “web first” = mixed-mode design, combining an initial invitation to a web survey with a subsequent phase of follow-up interviewing among the non-participants in the web survey – in this case: with interviewers conducting personal interviews*

Our analyses are based on the GGP pilot study 2018 (Emery et al., 2026), which offers us a unique source of data, given its experimental design (see Fig. 2). It was conducted in Germany, Croatia, and Portugal. The target population is defined as the residential population aged between 18 and 49 years. In each country, a probability sample was drawn. In Germany and Portugal, the sampling was regionally restricted. It was required that all samples included the rural population in a manner that was at least proportional to the target population, so that the conditions for online data collection were underestimated rather than overestimated. The German sample was drawn from 36 local residents’ registration offices in the federal state of Bavaria; 50% of the gross sample was recruited from communities with less than 50,000 inhabitants. The Portuguese sample was drawn in the Lisbon metropolitan area, with a quota of four predominantly urban and four predominantly rural areas, by random-route sampling, as no suitable sampling frame was available. In Croatia, a national sample was drawn, based on a national electoral register.

The study employs an experimental design. In each country, participants were randomly assigned to one of at least two experimental groups. The comparability of the two groups is statistically confirmed by matching (see Sect. 4.1). The reference group was interviewed in CAPI mode, in accordance with the design of GGS-I. However, in a few sections comprising sensitive questions, interviewers asked the respondents to fill out the questions themselves. In the experimental groups, a “web-first” design was employed: People were invited to participate in a web survey (CAWI) by providing a URL and a personalized identification code in an invitation letter. Those who did not participate were sent a first reminder letter after a period of two weeks, and a second reminder letter after a further two weeks. The second reminder announced that an interviewer would be visiting in order to conduct the interview in CAPI mode instead. After a few more days, the web option was closed and the CAPI follow-up interviews started, with the objective of converting non-respondents from the web survey into respondents.

In Portugal, the rural population was oversampled in the “web first” experimental group in order to achieve a more balanced distribution in the net sample, already expecting mode-specific selection effects (see Table 2). Furthermore, the face-to-face follow-ups in the “web-first” condition proved to be challenging in Portugal and were ultimately discontinued after only seven interviews. Within the Portuguese experimental group, we therefore use the CAWI cases only. In Germany, the follow-up process also remained incomplete, with only 42% of the target population remaining after the CAWI-phase being contacted by an interviewer, resulting in 340 face-to-face interviews. In all countries, the “web-first” experimental group therefore is primarily composed of web interviews.

In all experimental groups, unconditional pre-paid incentives were offered: 5 Euro cash in Germany, 40 Kuna cash in Croatia and a 5 Euro voucher in Portugal. Further country-specific experiments were conducted (Lugtig et al., 2022). The GGP pilot study of 2018 employed the GGS questionnaire, which had been redesigned for GGS-II, in its preliminary state at the time of the pilot’s implementation. This questionnaire version is published as an appendix in the documentation of the pilot study (Emery et al., 2026). In this questionnaire, we identified 23 indicators that are frequently employed in demographic research and that we utilize for the assessment of mode effects (see Table 1). Table 2 shows the number of completed interviews and the sociodemographic characteristics of each sub-sample.

### Methods

We approach the assessment of mode effects in four steps. Firstly, we collect evidence for mode-specific selection biases in the subsamples of the experimental groups. We do so by comparing the distributions of the basic sociodemographic facts, namely sex, age, nationality, education, and regional setting (see Table 2) in both the CAPI and “web-first” conditions. These variables should be easily reportable for all respondents and not affected by mode-measurement effects. We compare statistics separately for the three countries participating in the study.

**Fig. 3:**
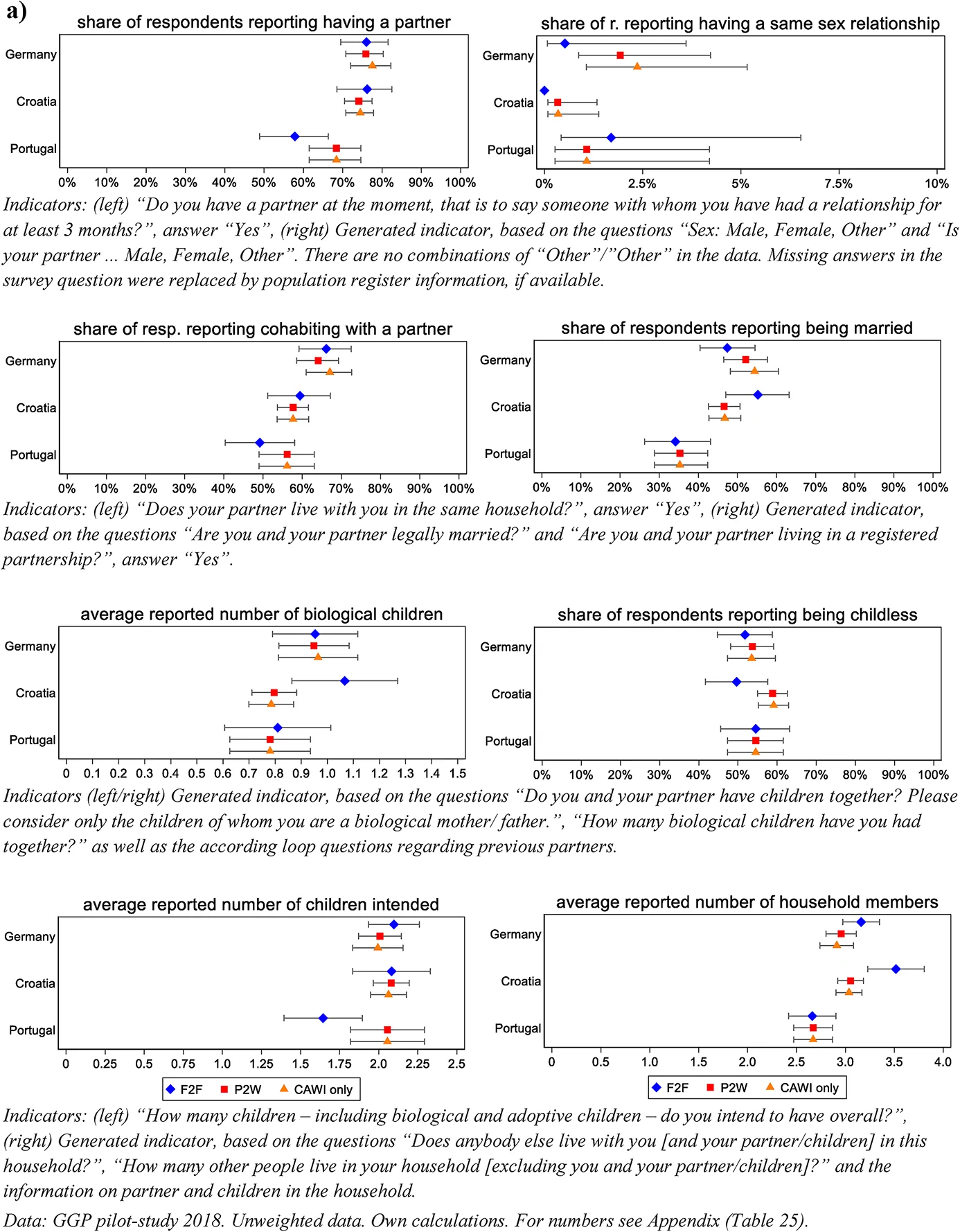

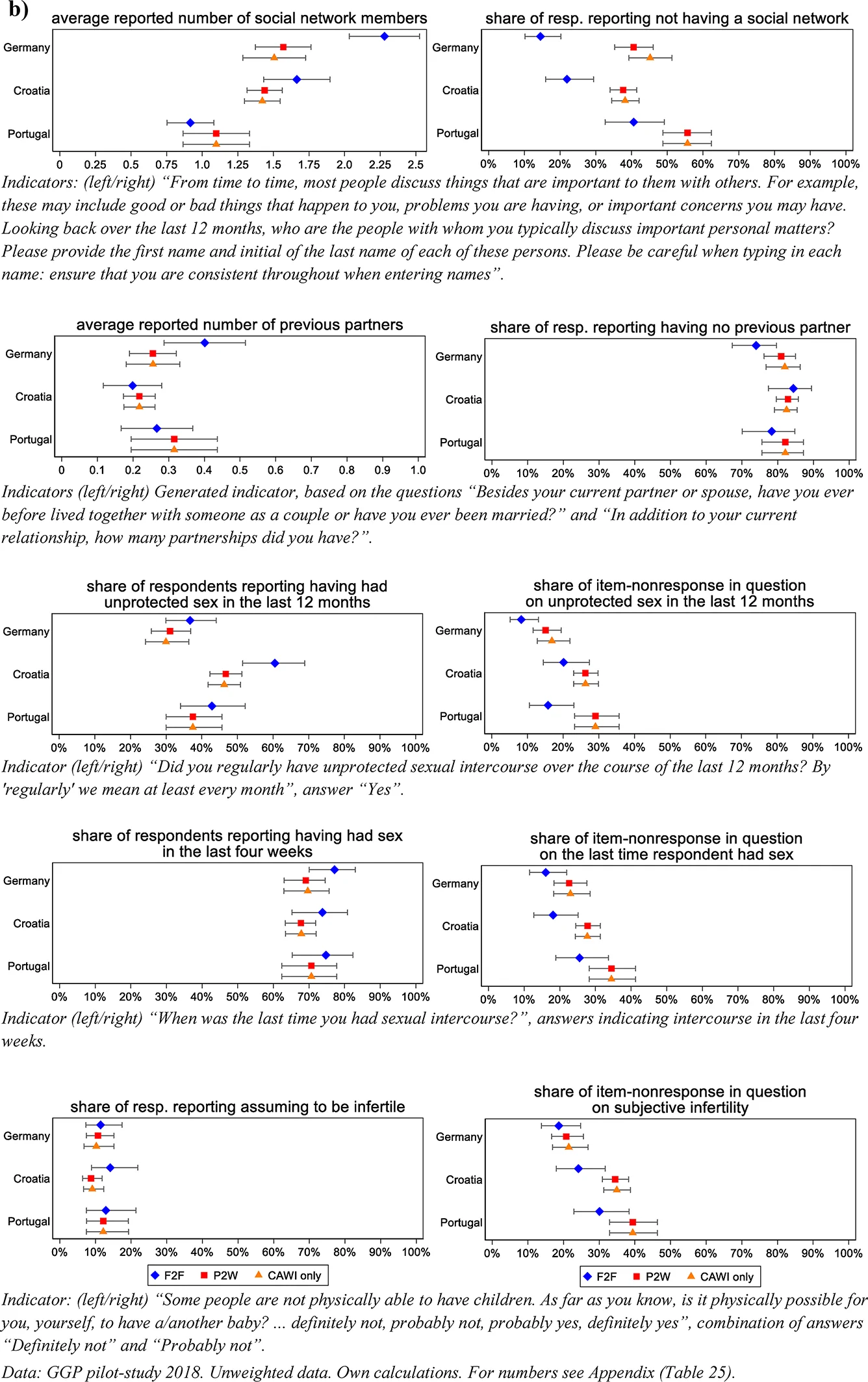

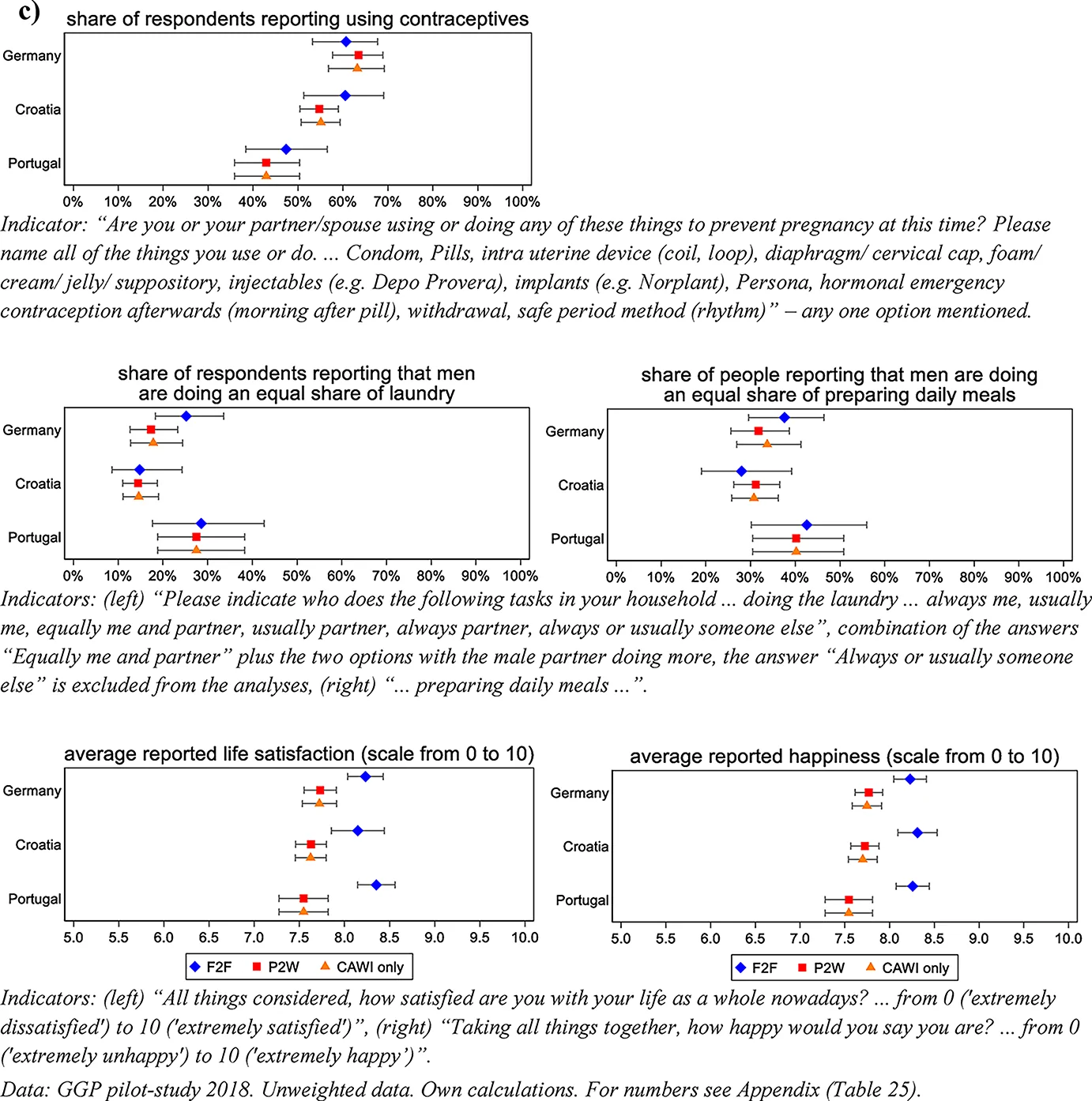
Mode differences for key demographic indicators (**a**) I, (**b**) II, (**C**) III

In a second step, we compare unweighted means and ratios of the 23 demographic key indicators, as presented in Table 1. By not weighting these descriptive analyses, differences in the mode-specific values represent the total mode effects and answer the question, in what way different modes will lead to different findings when measuring demographic key indicators without corrections (Fig. 3). However, the observed differences do not indicate the degree to which deviances or how different modes lead to different findings when demographic key indicators are measured with corrections by weights. These points are addressed in the next step. The weighted descriptive analyses (not shown) are highly similar to the unweighted results.

**Fig. 4:**
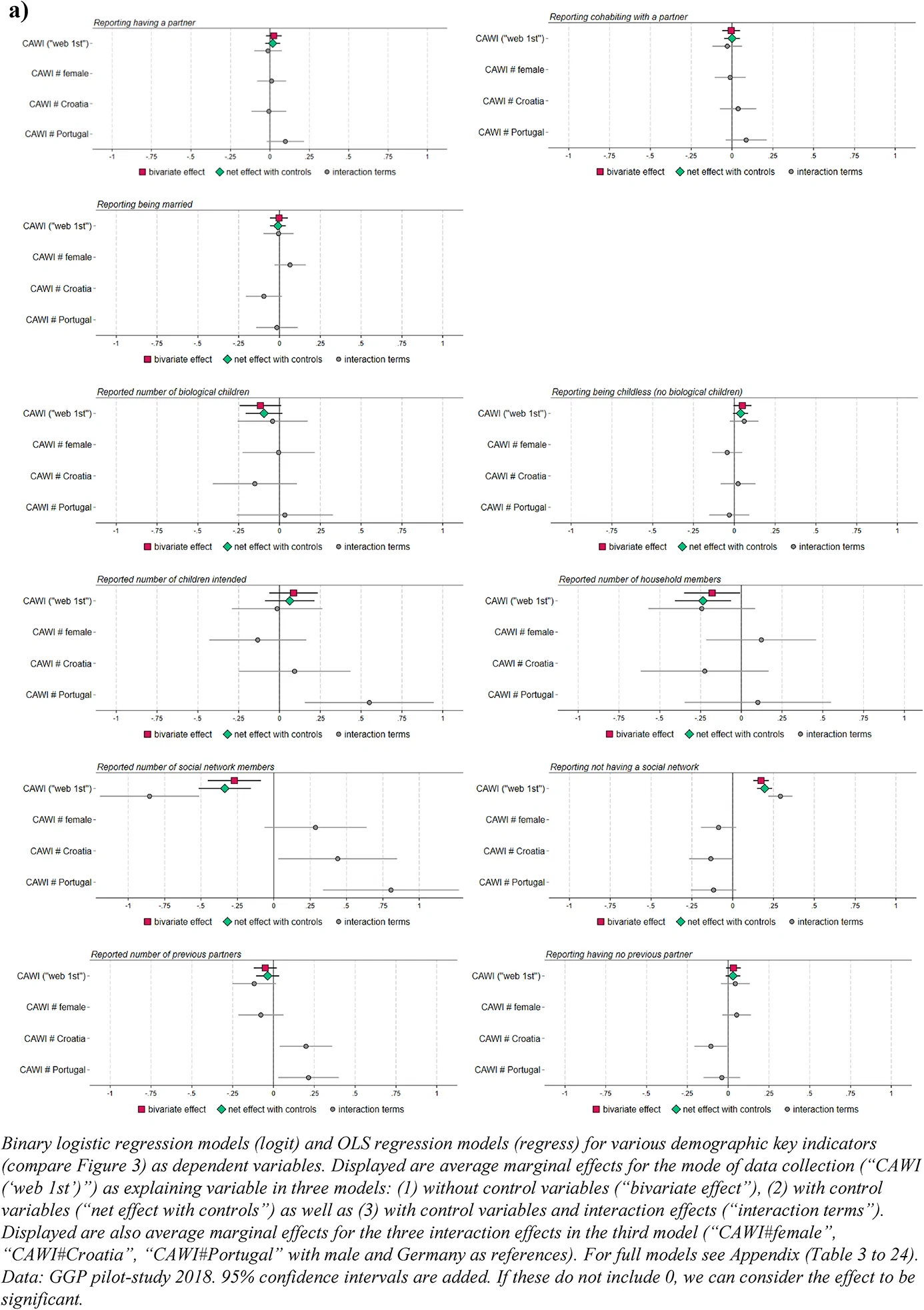

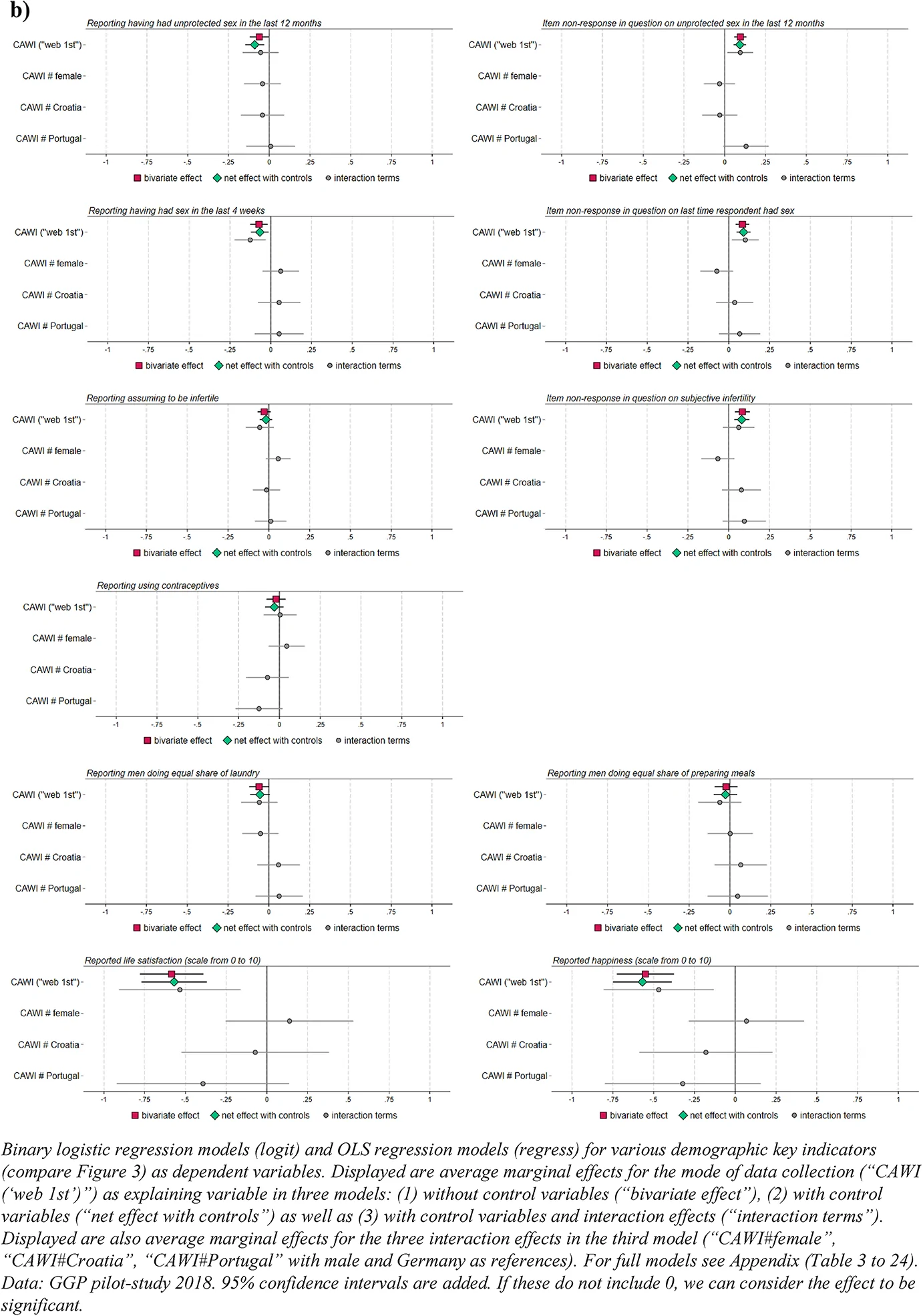
Effects of the mode on key demographic indicators (average marginal effects) (**a**) I, (**b**) II

In a third step, we perform regression analyses (Fig. 4 and tables in the appendix). We take the selected demographic key indicators as dependent variables and the mode of data collection (CAWI versus CAPI) as the independent variable. The sociodemographic indicators used in step one are added as control variables. We use binary logistic regression analyses for binary demographic indicators and linear OLS regression analyses for metric indicators. We interpret the effect of the mode in a bivariate regression as the total mode effect, just like the descriptive analyses in step 2. We interpret the net effect of the mode, when controlling for sample selectivity regarding sex, age, nationality, education, and region, as an approximation of the mode-measurement effect. These five control variables do not explain *all* possible selection effects present. In particular in Portugal and in Germany it is possible that sample compositions are affected by selectivity that is not fully controlled for, since here face-to-face follow-up interviews were discontinued before all web non-respondents had been contacted. Nevertheless, the five control variables are important predictors of the demographic outcome variables, and as such should correct for most of the mode-selection effects. We only use 22 out of the 23 demographic indicators in this step because the share of respondents in a same-sex relationship is too low to make valid statistical comparisons.

In a fourth and last step, we add interaction effects between the mode of data collection and two further characteristics to the regression models: the respondent’s sex and the country in which the survey was conducted. There are several further interactions which can be investigated. However, we focus on these two, which we consider particularly important given their central role in the design of the GGS. In extended and more exploratory analyses, we found no notable correlations for other indicators.

## Results

### Selection Effects

When comparing the experimental groups of the GGP pilot study, a few mode-selection effects are visible. Table 2 shows the distributions of socioeconomic indicators in the target population as well as in the samples (gross and net sample) for each experimental group (reference and experimental group) and for each country.

Within the gross samples, no significant differences between experimental groups can be found, with the exception of the oversampled rural population in Portugal. This indicates that the randomised assignment has generated very similar gross samples. This also has been confirmed by a Coarsened Exact Matching algorithm. The procedure identifies ‘statistical twins’ in the two experimental groups and tests whether the outcomes of analyses vary significantly if only the twins or if only the remaining unpaired cases are analysed. In this instance there was no significant difference between the two groups suggesting a robust randomization.

For evaluating mode-specific selection effects, the distributions in the net samples of the experimental groups (the three columns on the right of Table 2) need to be compared. Below each indicator, the outcome of a χ^2^-test is reported. If the level of significance is below p = 0.10 (10%) the distributions are highlighted in bold. We compare distributions for five indicators: sex, age, nationality, education, and regional setting. At the same time, we assess which mode leads to a distribution that is similar to the target population (left column) and/or to the according gross sample (columns in the centre). A detailed and more in-depth analysis can be found in Lugtig et al. (2022). 

**Table 2:** Sample description

		Target population^(1)^	Gross sample			Net sample		
			Total	Reference group	Exper. group	Reference group	Exper. group	Exper. group CAWI
		(in %)	(in %)	(in %)	(in %)	(in %)	(in %)	(in %)
Mode of data collection				CAPI	web 1st	CAPI	web 1st	CAWI
**Germany**								
* Sample size*			*2,256*	*720*	*1,536*	*193*	*318*	*261*
Sex	Male	51,6	51,2	51,3	51,2	47,7	48,4	47,5
	Female	48,4	48,8	48,7	48,8	52,3	51,6	52,5
				χ^2^ = 0.00; p = .972		χ^2^ = 0.03; p = .868		
Age	18–29	33,0	35,8	35,8	35,7	38,0	33,0	30,4
	30–39	28,0	30,8	30,8	30,8	29,2	30,1	32,3
	40–49/50	39,0	33,4	33,3	33,5	32,8	36,9	37,3
				χ^2^ = 0.00; p = .998		χ^2^ = 1.44; p = .486		
Nationality	German	90,7	79,6	78,5	80,1	88,1	87,7	88,8
	Non-German	9,3	20,4	21,5	19,9	11,9	12,3	11,2
				χ^2^ = 1.48; p = .478		χ^2^ = 0.20; p = .887		
Education ^(2)^	Lower education	38,1	— ^(6)^	— ^(6)^	— ^(6)^	**25,0**	**17,1**	12,3
	Medium educat	30,6	— ^(6)^	— ^(6)^	— ^(6)^	**26,6**	**32,9**	33,6
	Higher education	31,4	— ^(6)^	— ^(6)^	— ^(6)^	**48,4**	**50,0**	54,1
						**χ** ^**2**^ ** = 5.30; p = .071**		
Region. setting	Urban	50,0^(5)^	50,0 ^(5)^	50,0 ^(5)^	50,0 ^(5)^	**39,4**	**50,0**	53,6
	Rural	50,0 ^(5)^	50,0 ^(5)^	50,0 ^(5)^	50,0 ^(5)^	**60,6**	**50,0**	46,4
				χ^2^ = 0.00; p = 1.00		**χ** ^**2**^ ** = 5.46; p = .020**		
**Croatia**								
* Sample size*			*2,050*	*600*	*1,450*	*149*	*644*	*624*
Sex	Male	50,7	51,4	51,2	51,5	40,9	43,9	43,3
	Female	49,3	48,6	48,8	48,5	59,1	56,1	56,7
				χ^2^ = 0.02; p = .885		χ^2^ = 0.44; p = .505		
Age	18–29	34,6^(3)^	30,7	29,8	31,1	29,2	32,7	32,7
	30–39	33,1 ^(3)^	35,0	33,8	35,5	31,2	32,2	32,4
	40–49/50	32,4 ^(3)^	34,3	36,4	33,4	39,6	35,2	34,9
				χ^2^ = 1.58; p = .453		χ^2^ = 1.10; p = .577		
Nationality	Croatian	99,4 ^(3)^	— ^(6)^	— ^(6)^	— ^(6)^	99,3	99,8	99,8
	Non-Croatian	0,5 ^(3)^	— ^(6)^	— ^(6)^	— ^(6)^	0,7	0,2	0,2
						χ^2^ = 1.31; p = .253		
Education	Lower education	1,1 ^(3)^	— ^(6)^	— ^(6)^	— ^(6)^	**8,3**	**4,8**	4,8
	Medium educat	78,4 ^(3)^	— ^(6)^	— ^(6)^	— ^(6)^	**56,3**	**48,0**	47,2
	Higher education	20,3 ^(3)^	— ^(6)^	— ^(6)^	— ^(6)^	**35,4**	**47,2**	48,0
						**χ** ^**2**^ ** = 7.89; p = .019**		
Region. setting	Urban	— ^(4)^	62,5	62,2	62,6	**54,4**	**62,4**	63,8
	Rural	— ^(4)^	37,5	37,8	37,4	**45,6**	**37,6**	36,2
				χ^2^ = 0.04; p = .847		**χ** ^**2**^ ** = 3.30; p = .069**		
**Portugal**								
* Sample size*			*5,225*	*2,017*	*3,208*	*133*	*203 * ^*(7)*^	*203 * ^*(7)*^
Sex	Male	48,7 ^(3)^	— ^(6)^	— ^(6)^	— ^(6)^	41,2	39,2	39,2
	Female	51,4 ^(3)^	— ^(6)^	— ^(6)^	— ^(6)^	58,8	60,8	60,8
						χ^2^ = 0.12; p = .726		
Age	18–29	31,2 ^(3)^	— ^(6)^	— ^(6)^	— ^(6)^	36,4	36,7	36,7
	30–39	31,0 ^(3)^	— ^(6)^	— ^(6)^	— ^(6)^	28,9	27,7	27,7
	40–49/50	37,7^(3)^	— ^(6)^	— ^(6)^	— ^(6)^	34,7	35,6	35,6
						χ^2^ = 0.06; p = .969		
Nationality	Portuguese	93,7^(3)^	— ^(6)^	— ^(6)^	— ^(6)^	93,4	94,8	94,8
	Non-Portuguese	6,3 ^(3)^	— ^(6)^	— ^(6)^	— ^(6)^	6,6	5,2	5,2
						χ^2^ = 0.29; p = .588		
Education	Lower education	30,0 ^(3)^	— ^(6)^	— ^(6)^	— ^(6)^	18,2	10,5	10,5
	Medium educat	47,0 ^(3)^	— ^(6)^	— ^(6)^	— ^(6)^	49,6	54,7	54,7
	Higher education	23,1^(3)^	— ^(6)^	— ^(6)^	— ^(6)^	32,2	34,7	34,7
						χ^2^ = 3.72; p = .156		
Region. setting	Urban	— ^(4)^	67,1	**83,8**	**56,4**	37,7	39,9	39,9
	Rural	— ^(4)^	32,9	**16,2**	**43,6**	62,3	60,1	60,1
				**χ** ^**2**^ ** = 415.6; p = .000**		χ^2^ = 0.16; p = .687		

Remarks: If differences between experimental groups are tested as significant below p = .01 (10%) then the according distributions are highlighted in bold

(1) Information for the target population are based on official statistics

(2) For Germany, the educational classification is based solely on the highest level of school education attained (not including vocational training additionally, as the ISCED for Germany requires), since reference data from the target population are only available for school educational level

(3) For Croatia, only information for sex and age is available for people aged 18–49 years; for other indicators it is available for people aged 20–49 years only

For Portugal, all information about the target population refers to people aged 20–49 years

(4) Information on the distribution between urban and rural areas is not available for Croatia and Portugal

(5) For Germany, the distribution between urban and rural areas is 50% each by definition, due to the application of a quota

(6) For Germany, education is not known for the gross sample because it is not provided by population registers

For Croatia, education and nationality are not known for the gross sample

For Portugal, no information on the gross sample is available except the regional setting because a random-route sampling and a regional quota were applied

(7) Due to sampling design, there were difficulties in carrying out the face-to-face follow-up in the “web-first” sample

Therefore, we only include the CAWI cases and the face-to-face cases from the reference group

There are few mode-selection effects in the GGS pilot study. The strongest effect is a mode-specific educational bias: People with a low level of education are significantly underrepresented across all modes. However, they are more underrepresented among the online participants in the “web-first” experimental group than among the face-to-face interviews in the CAPI reference group. This is true in all three countries.

Regarding other socioeconomic indicators, the experimental group tends to show greater similarity to the target population than the reference group. This is particularly true for the ratio of urban versus rural regions in Germany and in Croatia, which is the second strongest mode-selection effect. In the reference group, the interviewers were less successful in recruiting people in urban regions than they were in rural regions, leading to a significant regional bias. In the experimental groups, this is not the case. In Portugal, the same mode-selection effect is visible. Here, the regional bias concerns both experimental groups, but is stronger in the CAPI reference group than in the “web first” experimental group. In the net samples in Portugal, the distributions are about equal because the rural regions had been over-sampled in the “web first” experimental group.

Regarding the age structure, diverging and insignificant mode-selection differences are visible: The “web-first” sample is slightly younger in Croatia and somewhat older in Germany than the CAPI sample. In Portugal, there is no difference. We cannot detect relevant biases or selection effects regarding citizenship. Biases by sex are similar in both modes, with women being slightly over-represented, so that no mode-specific selection effect is visible.

The results of the mode-selection effects are largely consistent with those reported in existing literature (Watson & Wooden, 2009). It is possible that they may cause or increase mode-specific differences in the measured distributions of demographic indicators, particularly if the data are not weighted. As is the case for all forms of nonresponse bias, weighting is required to ensure that analytical samples are representative of the target population.

### Mode Differences in Descriptive Analyses

We assess the overall mode effects by comparing the unweighted ratios and means of the demographic key indicators classified according to experimental group and mode. The following Figures (Fig. 3a to 3c) show three values for each indicator, differentiated by country: one value for the CAPI reference group, one value for the entire “web-first” experimental group, as well as a third value for the CAWI respondents within that experimental group. The Figures show 95% confidence intervals. Due to the relatively low number of cases, confidence intervals tend to be large, and substantial mode effects are often hard to identify.

The ratios and means should not be interpreted demographically. In order to obtain meaningful results, the indicators would have to be calculated for specific sub-groups, e.g. childlessness for women aged 45 to 50. In the following analyses, however, *all* available cases are used for all indicators, as case numbers would be too small for any statistical analyses otherwise.

Overall mode effects cannot be generalised. They vary greatly between indicators. Often, they also vary greatly between countries. An example of a mode effect that is robust across all three countries is that respondents in CAWI mode are more likely to report having zero social network members than those in the CAPI reference group. This finding is in line with our expectations and could be interpreted as an effect of satisficing strategies that are more pronounced when no interviewer is present. Further examples are that CAPI respondents report higher life satisfaction and more happiness than CAWI respondents, which is in line with our assumption of higher perceived social pressure when an interviewer is present.

The findings reveal that the mode of collecting data in a survey may very well affect the measurement of demographic indicators. However, this is not always the case. Mode effects occur to various extents and in diverging directions, depending on the indicator as well as on country contexts.

### Mode Effects and Interaction Effects in Regression Models

The mode differences presented in Fig. 3a to 3c are more or less all confirmed by the regression models (Fig. 4a and 4b). The full models, including control variables, can be found in the appendix. For a better overview, the following figures present the effect of the mode graphically, without revealing the effects of the control variables. The red square marks the effect of the mode on the demographic key indicators in a bivariate regresssion, without any control variable. The green diamond marks the net mode effect in multivariate regression models after controlling for sex, age, nationality, education, and region. The grey circle marks the effect of the mode and of the interaction terms in a third model that additionally includes three interaction terms.

Given that the selection effects are to a considerable degree controlled for in the multivariate models, the net effects for the mode (green diamond) display the approximate mode-specific measurement effects. Based on these, we can assess whether the measurement effects are in line with our theoretical expectations (see Table 1). Based on the comparison of net effect (green diamond) and “bivariate effect” in models without controls (red square), we can assess the ways in which sample selectivity affects the measurement of the 22 analysed demographic indicators.

For selection effects in the measurement of the demographic key indicators, we find no evidence. There are more or less two indicators for which controlling for selectivity increases the mode differences visibly (the number of household members and the number of social network members). However, these differences are insignificant, very small and probably random errors. Despite the mode-specific selectivity regarding education and regional setting (see Table 2), none of the 22 demographic indicators we are analysing seems to be affected by this selectivity.

Regarding the measurement effects, the findings are in accordance with the descriptive results presented in the previous section: Mode-specific differences occur. However, the occurrence, extent and direction are specific for each indicator and partly also dependent on the country context. Again, we find evidence for two of our assumptions: Mode effects may occur due to a stronger satisficing bias in the self-administered CAWI mode, for example regarding the number of social network members or the number of household members. Effects also may occur due to a stronger social desirability bias in CAPI mode, when an interviewer is present, e.g. regarding life satisfaction and happiness. We do not find these expectations confirmed for every indicator. We also do not find evidence for any mode-specific recency or primacy effects, as we had expected for the questions regarding contraceptive use and subjective infertility.

## Discussion

### Summary of Results

In this article, we investigated the consequences of survey mode changes for the comparability of demographic key indicators. We exploited unique data of a GGP mode experiment, conducted in Germany, Croatia, and Portugal. In all countries, we found a few mode-selection effects as well as mode-measurement effects.

We found quite strong overall mode differences when we compared unweighted means and ratios for the CAWI and CAPI respondents. We found strong mode-selection effects for the educational level and regional setting of respondents, with higher educated people being more likely to participate in the CAWI and people in rural regions being more likely to participate in the CAPI mode. After controlling for selection effects, we still found nearly the identical mode differences among the 22 selected demographic key indicators, indicating the likely presence of mode-measurement effects. These are in line with two of our expectations, offering two plausible ways in which the mode effects can be interpreted. First, biases due to social desirability occurred when there were norms defining a certain answer as socially expected; these were stronger in CAPI mode, when an interviewer was present. Second, biases occurred that could be due to satisficing when answering a question implied a high respondent burden; these biases were stronger in the self-administered CAWI mode.

Effects that may be due to social desirability were visible for well-being as well as for sexual activity. This encompassed four indicators for life satisfaction and happiness, as well as for unprotected sexual intercourse in the past 12 months and sexual intercourse in the past 4 weeks. In all cases, the average value for CAWI was lower, which may indicate a greater tendency to provide socially undesirable responses. The effects for life satisfaction and happiness were particularly pronounced, which may be attributable to the absence of a switch to a self-administered mode with interviewers handing over their computer to the respondents. However, the indicators on sexual activity also showed significant effects in the regression models. This suggests that the strategy to make respondents fill in their answers to sensitive questions on their own is unable to fully eliminate the social pressure experienced due to the physical presence of an interviewer.

In the case of further indicators for which we had anticipated socially desirable responses, no significant effects were identified. This included the proportion of men contributing to household chores. This may be attributed to the limited social pressure to distribute housework equally or to the fact that we were unable to focus on male respondents due to low case numbers. For the number of children intended, the only significant effect was an interaction for CAWI mode in Portugal. This could indicate that unfulfilled fertility desires are less socially accepted in Portugal than in Germany. Remarkably, we did not find any effects for contraceptive use or for subjective infertility. Despite respondents filling in their answers to these questions on their own, instead of the interviewer, this finding is unexpected and requires further examination.

Effects that could be interpreted as due to satisficing were visible for complicated questions as well as for questions that activated loops, for answers as well as for item nonresponse. The most pronounced effect concerned the number of social network members, as well as the reporting of no social network at all: In CAWI, far fewer network members were reported. This reduced the effort required to read the lengthy question. It reduced the cognitive effort to decide upon the correct answer. And it reduced the effort required to type in names in an uncommon questionnaire tool. In CAWI, also fewer household members were reported, which reduced the number of loops that followed.

Furthermore, effects were visible for item-nonresponse in the questions on unprotected sexual intercourse in the past 12 months, on the last time respondents had sexual intercourse, and on subjective infertility. The proportions were higher in CAWI, which presumably reduced the cognitive effort of choosing the most appropriate answer category. In the case of these indicators, we had contradicting expectations: On the one hand, it seemed plausible that CAWI respondents reduced their effort by skipping these questions more often than CAPI respondents. On the other hand, it was imaginable that social desirability would lead to a higher item nonresponse in CAPI mode. If the interpretation holds, the presumable effect of satisficing was more pronounced than that of social desirability. A reason could be that the strategy to make respondents fill in their answers to sensitive questions on their own in the CAPI interviews weakened the social desirability effects.

Remarkably, two further expected effects related to loops were insignificant: the number of biological children, including the prevalence of childlessness, as well as the number of previous partners. We only found a significant interaction effect on the number of previous partners in Germany; and we found comparably strong mode differences regarding the number of biological children in Croatia. It is unclear why one indicator activating loops was significant while two others were not.

Neither in the descriptive findings nor in the regression models did we find evidence for primacy effects (in CAWI) or recency effects (in CAPI). There were two indicators for which we expected such effects: contraceptive use and subjectively perceived infertility. It may be the case that these were not decently susceptible to primacy or recency effects. However, alternative indicators had not been included in the GGS questionnaire.

Only three significant interaction effects were identified in relation to the country in which the study was conducted. In addition to the two aforementioned affects, this concerned the number of social network members, which was reported to be lower by CAWI respondents, particularly in Germany. This could indicate, for instance that German respondents were particularly irritated by the lengthy question. Or they were more hesitant to provide names due to concerns about data protection. It is evident that the country context has the potential to influence results. It is possible that cultural context may modify social desirability effects: Social norms are likely to vary between the countries, at least in details. Accordingly, an answer that is perceived as socially (un)desirable in one country may be less so in another, resulting in missing or more moderate mode effects. As a tendency, we find stronger effects related to social desirability in Portugal (e.g. regarding life satisfaction, happiness, children intended, item nonresponse) and weaker effects in Germany, compared to Croatia. Satisficing effects might vary for cultural or technical reasons. For instance, there may be more people feeling time pressure and therefore being prone to speeding in one society than in another. Also, there may be stronger or weaker norms regarding the duty to answer accurately in scientific surveys. As a tendency, we find stronger effects related to satisficing in Germany (e.g. regarding the size of the social network, number of previous partners) and weaker effects in Portugal, compared to Croatia. We can imagine more complex interactions with the country context that need further investigation, such as the incentive of 40 Kuna in Croatia having a stronger impact than that of 5 Euro in Germany.

There was no compelling evidence to suggest an interaction between the mode and gender, but a few insignificant effects. For example, women tended to report a greater number of social network members in CAWI mode. This may indicate that the presumable satisficing effect was less pronounced for them. However, the role of gender or other indicators as moderating effects requires further investigation.

Against the state of research on mode effects, our results are unsurprising in two senses: First, effects due to satisficing as well as due to social desirability are common (Bowling, 2005; Galesic & Bosnjak, 2009; Holbrook & Krosnick, 2010; Holbrook et al., 2003; Kreuter et al., 2008; Preisendörfer & Wolter, 2014). Second, effects vary remarkably between specific indicator and coutry contexts (Heerwegh, 2009; Klausch et al., 2013). Because of this variation, our research contributes to the state of research by providing evidence regarding 22 specific key indicators in demographic research. Compared to research that explores closely related research fields, our findings are partly expectable, such as the strong effects for happiness and life-satisfaction. These compare to similar findings regarding partnership satisfaction, also based on GGS data (Schumann & Lück, 2023). Partly, our findings are rather surprising such as the absence of effects regarding the number of biological children and other loop questions, since these had been found to be prone to satisficing in earlier GGS data (Kreuter et al., 2008; Ruckdeschel et al., 2016).

### Limitations

This pilot study and this analysis inevitably had limitations. One limitation concerned the relatively low case numbers. These lead to several insignificant effects, making it hard to distinguish between substantial findings and random errors. They also made it necessary to analyse all demographic indicators, using the full samples, instead of reducing analyses to meaningful subsamples, which is the standard practice in empirical demographic research. The selection of available indicators had an impact on our findings. In particular, the GGP pilot study does not provide suitable indicators for identifying primacy or recency effects. In future GGS studies, this could be done e.g. by incorporating indicators with many answer categories and randomized answer orders. Our analyses also lacked meaningful paradata to confirm the presumed higher prevalence of satisficing in CAWI mode. Furthermore, this research was limited by the number of countries included. The three countries showed a large variety of preconditions for survey research. Still, it is not possible to generalize for other countries with certainty. Generally, the country context as well as other meaningful contexts that might mediate findings could be elaborated in much greater depths, which could not be done in this research due to limited time and space.

### Conclusions

The unique data, derived from a three-country mode experiment utilizing the GGS questionnaire, enabled insights into a central methodological question in contemporary empirical demographic research that were previously unfeasible. A mode shift within the Generations and Gender Survey, as an ongoing longitudinal study, as well as mode differences between data collections in different countries challenge the comparability between datasets. Similar challenges are or will be found globally in most panels and survey programmes, since the transition from face-to-face interviewing to self-administered online interviews as the predominant mode of data-collection is universal (Carpenter, 2017; Davern et al., 2021; Gummer et al., 2020; Wolf et al., 2021). For the time of the transition, it also challenges any cross-national comparative research, as soon as modes deviate across countries. This will soon impact panels that had been assumed to be unable to rely on web interviewing due to coverage problems, such as SHARE with elder people as target population (Bruijne & Kalwij, 2024). In all these panels and survey programmes the comparability between datasets is questioned by this process, and empirical knowledge is needed to even assess the extent of the problem. This paper contributes to this knowledge with a broad overview on key indicators in demographic research. For other panels and research fields it may serve as a template. However, its conclusions will very likely hold for any panel in any field of research.

The findings presented in this paper have several implications for primary researchers. Some concern the questionnaire design: Whenever possible, influences due to social desirability should be avoided and questions involving a high respondent burden should be simplified. When conducting a CAPI survey, mode switches for sensitive questions with respondents filling in their answers on their own (“CASI” mode) should be used, since this reduces the social desirability effects. It is recommended that loops be kept brief or avoided all together. Generally, all measures should be taken to avoid all kinds of biases in measurement.

These recommendations are true for every survey or panel, independent from how many and which survey modes are used since biases are generally undesirable. Furthermore, they are helpful for making survey data comparable to other surveys, since measurement differences between modes often occur with a particular bias being more pronounced in one mode than in another. The fewer questions in a survey are prone to social desirability or survey satisficing or to other sources of biases, the fewer mode effects are likely to occur. This concerns studies that are using more than one mode in particular, since any mode-difference will be immanent in their data and in all analyses. However, it also concerns other surveys and panels since findings based on their data may be compared to findings based on other data.

Data users must be aware of the survey design and of the modes used in a study. When using data collected in various modes, either in a longitudinal study or in a cross-national comparative study, the mode should always be controlled for. Mode differences should be considered when interpreting findings. Also, when analysing data from a survey or panel that uses one single mode only, this mode needs to be reflected when interpreting findings. If a measured value seems surprising this may be the case because expectations were based on previous research, conducted in a different mode. Also, each mode is prone to specific biases; and data users should be aware of them. The knowledge regarding common patterns of biases and mode effects, as outlined in this paper, is beneficial in that respect. Such knowledge will become of increasing relevance as the shift towards CAWI continues across surveys used in demography and elsewhere, irrespective of the impact such a shift has on the data collected and its comparability with long standing data sources.

## Supplementary Information

Below is the link to the electronic supplementary material.

Supplementary file1 (DOCX 98 kb)

Supplementary file2 (JPG 380 kb)

## Data Availability

The dataset generated and analysed by the survey research during the current study are available in the "GGP Data Portal" (https://www.ggp-i.org), after registration and login, as “GGP Push to Web Pilot”.
