# Supplementary material for: How do Modes of Data Collection Affect the Measurement of Demographic Key Indicators? Findings from a Three-Country Experiment Using the Generations and Gender Survey
Source: Eur J Popul. 2026 Aug 13;42(1):22. doi: 10.1007/s10680-026-09777-7 (PMC13490029; doi:10.1007/s10680-026-09777-7)
Supplement: Supplementary file 1 [file 10680_2026_9777_MOESM1_ESM.docx]

***Appendix: Full Regression Models***

**Table 3 Effect of mode on respondents reporting having a partner (logit model, average marginal effects)**

|  | Model 1 | Model 2 | Model 3 |
| --- | --- | --- | --- |
| “web 1st” group *(ref.: CAPI)* | 0.025 | 0.018 | -0.012 |
|  | (se=0.025) | (se=0.024) | (se=0.045) |
|  | [-0.025 \| 0.074] | [-0.030 \| 0.065] | [-0.100 \| 0.075] |
| female *(ref.: male)* |  | 0.090*** | 0.082* |
|  |  | (se=0.022) | (se=0.040) |
|  |  | [0.047 \| 0.133] | [0.004 \| 0.160] |
| age: 18-30 *(ref.: 31-40)* |  | -0.292*** | -0.292*** |
|  |  | (se=0.027) | (se=0.027) |
|  |  | [-0.346 \| -0.239] | [-0.346 \| -0.239] |
| age: 41-50 *(ref.: 31-40)* |  | 0.022 | 0.023 |
|  |  | (se=0.024) | (se=0.024) |
|  |  | [-0.024 \| 0.069] | [-0.024 \| 0.069] |
| education: low *(ref.: medium)* |  | -0.030 | -0.030 |
|  |  | (se=0.038) | (se=0.038) |
|  |  | [-0.105 \| 0.044] | [-0.104 \| 0.045] |
| education: high *(ref.: medium)* |  | -0.004 | -0.003 |
|  |  | (se=0.023) | (se=0.023) |
|  |  | [-0.050 \| 0.042] | [-0.049 \| 0.043] |
| no national citizenship *(ref.: nat.)* |  | 0.064 | 0.068 |
|  |  | (se=0.048) | (se=0.048) |
|  |  | [-0.030 \| 0.159] | [-0.025 \| 0.161] |
| region: rural *(ref.: urban)* |  | 0.029 | 0.027 |
|  |  | (se=0.022) | (se=0.022) |
|  |  | [-0.015 \| 0.072] | [-0.016 \| 0.071] |
| country: Croatia *(ref.: Germany)* |  | -0.026 | -0.016 |
|  |  | (se=0.025) | (se=0.045) |
|  |  | [-0.076 \| 0.023] | [-0.104 \| 0.073] |
| country: Portugal *(ref.: Germany)* |  | -0.122*** | -0.188*** |
|  |  | (se=0.032) | (se=0.051) |
|  |  | [-0.185 \| -0.059] | [-0.288 \| -0.088] |
| “web 1st” group # female |  |  | 0.011 |
|  |  |  | (se=0.047) |
|  |  |  | [-0.081 \| 0.103] |
| “web 1st” group # Croatia |  |  | -0.007 |
|  |  |  | (se=0.056) |
|  |  |  | [-0.117 \| 0.104] |
| “web 1st” group # Portugal |  |  | 0.098 |
|  |  |  | (se=0.061) |
|  |  |  | [-0.021 \| 0.217] |
| McFadden R^2^ | 0.001 | 0.111*** | 0.113*** |
| corr. McFadden R^2^ | -0.003 | 0.090*** | 0.089*** |
| Count R^2^ | 0.728 | 0.747*** | 0.753*** |
| corr. Count R^2^ | 0.000 | 0.069*** | 0.089*** |
| degrees of freedom (df) | 1 | 10 | 13 |
| Observations | 1,491 | 1,491 | 1,491 |

Displayed are average marginal effects, standard errors and 95% confidence intervals. † p<0.10,
* p<0.05, ** p<0.01, *** p<0.001. Data: GGP Pilot Study 2018. Wording of dependent variable:
“Do you have a partner at the moment, that is to say someone with whom you have had a relationship for at least 3 months?”, answer “Yes”. Source: own computation.

**Table 4 Effect of mode on respondents reporting cohabiting with a partner (logit model, average marginal effects)**

|  | Model 1 | Model 2 | Model 3 |
| --- | --- | --- | --- |
| “web 1st” group *(ref.: CAPI)* | -0.005 | 0.000 | -0.029 |
|  | (se=0.028) | (se=0.025) | (se=0.047) |
|  | [-0.060 \| 0.049] | [-0.048 \| 0.049] | [-0.120 \| 0.062] |
| female *(ref.: male)* |  | 0.063** | 0.070^†^ |
|  |  | (se=0.022) | (se=0.041) |
|  |  | [0.019 \| 0.106] | [-0.010 \| 0.150] |
| age: 18-30 *(ref.: 31-40)* |  | -0.488*** | -0.487*** |
|  |  | (se=0.027) | (se=0.027) |
|  |  | [-0.541 \| -0.435] | [-0.540 \| -0.434] |
| age: 41-50 *(ref.: 31-40)* |  | 0.037 | 0.038 |
|  |  | (se=0.027) | (se=0.027) |
|  |  | [-0.016 \| 0.090] | [-0.015 \| 0.091] |
| education: low *(ref.: medium)* |  | -0.029 | -0.029 |
|  |  | (se=0.038) | (se=0.038) |
|  |  | [-0.103 \| 0.045] | [-0.103 \| 0.046] |
| education: high *(ref.: medium)* |  | -0.022 | -0.022 |
|  |  | (se=0.024) | (se=0.024) |
|  |  | [-0.069 \| 0.025] | [-0.069 \| 0.025] |
| no national citizenship *(ref.: nat.)* |  | 0.030 | 0.032 |
|  |  | (se=0.053) | (se=0.053) |
|  |  | [-0.074 \| 0.134] | [-0.072 \| 0.136] |
| region: rural *(ref.: urban)* |  | 0.027 | 0.026 |
|  |  | (se=0.023) | (se=0.023) |
|  |  | [-0.018 \| 0.071] | [-0.019 \| 0.070] |
| country: Croatia *(ref.: Germany)* |  | -0.083** | -0.105* |
|  |  | (se=0.026) | (se=0.047) |
|  |  | [-0.135 \| -0.031] | [-0.196 \| -0.013] |
| country: Portugal *(ref.: Germany)* |  | -0.120*** | -0.172*** |
|  |  | (se=0.032) | (se=0.050) |
|  |  | [-0.183 \| -0.057] | [-0.271 \| -0.074] |
| “web 1st” group # female |  |  | -0.011 |
|  |  |  | (se=0.048) |
|  |  |  | [-0.106 \| 0.084] |
| “web 1st” group # Croatia |  |  | 0.037 |
|  |  |  | (se=0.057) |
|  |  |  | [-0.074 \| 0.149] |
| “web 1st” group # Portugal |  |  | 0.087 |
|  |  |  | (se=0.064) |
|  |  |  | [-0.039 \| 0.213] |
| McFadden R^2^ | 0.000 | 0.199*** | 0.200*** |
| corr. McFadden R^2^ | -0.003 | 0.181*** | 0.179*** |
| Count R^2^ | 0.591 | 0.759*** | 0.759*** |
| corr. Count R^2^ | 0.000 | 0.411*** | 0.411*** |
| degrees of freedom (df) | 1 | 10 | 13 |
| Observations | 1,482 | 1,482 | 1,482 |

Displayed are average marginal effects, standard errors and 95% confidence intervals. † p<0.10,
* p<0.05, ** p<0.01, *** p<0.001. Data: GGP Pilot Study 2018. Wording of dependent variable: “Does your partner live with you in the same household?”, answer “Yes”. Source: own computation.

**Table 5 Effect of mode on respondents reporting being married (logit model, average marginal effects)**

|  | Model 1 | Model 2 | Model 3 |
| --- | --- | --- | --- |
| “web 1st” group *(ref.: CAPI)* | -0.003 | -0.009 | -0.006 |
|  | (se=0.028) | (se=0.025) | (se=0.047) |
|  | [-0.058 \| 0.052] | [-0.058 \| 0.039] | [-0.098 \| 0.087] |
| female *(ref.: male)* |  | 0.034 | -0.012 |
|  |  | (se=0.022) | (se=0.041) |
|  |  | [-0.010 \| 0.078] | [-0.091 \| 0.068] |
| age: 18-30 *(ref.: 31-40)* |  | -0.455*** | -0.458*** |
|  |  | (se=0.027) | (se=0.027) |
|  |  | [-0.507 \| -0.403] | [-0.510 \| -0.406] |
| age: 41-50 *(ref.: 31-40)* |  | 0.124*** | 0.120*** |
|  |  | (se=0.031) | (se=0.031) |
|  |  | [0.064 \| 0.184] | [0.060 \| 0.180] |
| education: low *(ref.: medium)* |  | -0.026 | -0.024 |
|  |  | (se=0.037) | (se=0.037) |
|  |  | [-0.099 \| 0.047] | [-0.097 \| 0.049] |
| education: high *(ref.: medium)* |  | -0.020 | -0.018 |
|  |  | (se=0.024) | (se=0.024) |
|  |  | [-0.067 \| 0.027] | [-0.065 \| 0.029] |
| no national citizenship *(ref.: nat.)* |  | 0.046 | 0.046 |
|  |  | (se=0.053) | (se=0.052) |
|  |  | [-0.057 \| 0.149] | [-0.056 \| 0.149] |
| region: rural *(ref.: urban)* |  | 0.040^†^ | 0.040^†^ |
|  |  | (se=0.023) | (se=0.023) |
|  |  | [-0.005 \| 0.085] | [-0.005 \| 0.085] |
| country: Croatia *(ref.: Germany)* |  | -0.027 | 0.041 |
|  |  | (se=0.027) | (se=0.048) |
|  |  | [-0.080 \| 0.025] | [-0.053 \| 0.136] |
| country: Portugal *(ref.: Germany)* |  | -0.151*** | -0.140** |
|  |  | (se=0.032) | (se=0.050) |
|  |  | [-0.214 \| -0.088] | [-0.238 \| -0.042] |
| “web 1st” group # female |  |  | 0.065 |
|  |  |  | (se=0.049) |
|  |  |  | [-0.031 \| 0.160] |
| “web 1st” group # Croatia |  |  | -0.095^†^ |
|  |  |  | (se=0.056) |
|  |  |  | [-0.206 \| 0.015] |
| “web 1st” group # Portugal |  |  | -0.016 |
|  |  |  | (se=0.065) |
|  |  |  | [-0.143 \| 0.111] |
| McFadden R^2^ | 0.000 | 0.220*** | 0.222*** |
| corr. McFadden R^2^ | -0.003 | 0.202*** | 0.201*** |
| Count R^2^ | 0.539 | 0.737*** | 0.740*** |
| corr. Count R^2^ | 0.000 | 0.430*** | 0.437*** |
| degrees of freedom (df) | 1 | 10 | 13 |
| Observations | 1,479 | 1,479 | 1,479 |

Displayed are average marginal effects, standard errors and 95% confidence intervals. † p<0.10,
* p<0.05, ** p<0.01, *** p<0.001. Data: GGP Pilot Study 2018. Dependent variable: generated indicator, based on the questions “Are you and your partner legally married?” and “Are you and your partner living in a registered partnership?”, answer “Yes”. Source: own computation.

**Table 6 Effect of mode on reported number of biological children (OLS regression, average marginal effects)**

|  | Model 1 | Model 2 | Model 3 |
| --- | --- | --- | --- |
| “web 1st” group *(ref.: CAPI)* | -0.117^†^ | -0.095^†^ | -0.042 |
|  | (se=0.065) | (se=0.057) | (se=0.109) |
|  | [-0.244 \| 0.011] | [-0.207 \| 0.018] | [-0.257 \| 0.173] |
| female *(ref.: male)* |  | 0.176*** | 0.179^†^ |
|  |  | (se=0.052) | (se=0.095) |
|  |  | [0.075 \| 0.278] | [-0.007 \| 0.364] |
| age: 18-30 *(ref.: 31-40)* |  | -0.967*** | -0.966*** |
|  |  | (se=0.062) | (se=0.062) |
|  |  | [-1.088 \| -0.845] | [-1.088 \| -0.845] |
| age: 41-50 *(ref.: 31-40)* |  | 0.352*** | 0.348*** |
|  |  | (se=0.064) | (se=0.064) |
|  |  | [0.226 \| 0.477] | [0.223 \| 0.474] |
| education: low *(ref.: medium)* |  | 0.164^†^ | 0.171* |
|  |  | (se=0.087) | (se=0.087) |
|  |  | [-0.006 \| 0.334] | [0.000 \| 0.341] |
| education: high *(ref.: medium)* |  | -0.115* | -0.110* |
|  |  | (se=0.056) | (se=0.056) |
|  |  | [-0.224 \| -0.005] | [-0.219 \| -0.000] |
| no national citizenship *(ref.: nat.)* |  | 0.376** | 0.377** |
|  |  | (se=0.123) | (se=0.123) |
|  |  | [0.135 \| 0.617] | [0.136 \| 0.618] |
| region: rural *(ref.: urban)* |  | 0.105* | 0.105* |
|  |  | (se=0.053) | (se=0.053) |
|  |  | [0.001 \| 0.209] | [0.001 \| 0.209] |
| country: Croatia *(ref.: Germany)* |  | -0.005 | 0.108 |
|  |  | (se=0.062) | (se=0.112) |
|  |  | [-0.127 \| 0.117] | [-0.113 \| 0.328] |
| country: Portugal *(ref.: Germany)* |  | -0.141^†^ | -0.159 |
|  |  | (se=0.075) | (se=0.117) |
|  |  | [-0.288 \| 0.006] | [-0.390 \| 0.071] |
| “web 1st” group # female |  |  | -0.005 |
|  |  |  | (se=0.113) |
|  |  |  | [-0.226 \| 0.216] |
| “web 1st” group # Croatia |  |  | -0.151 |
|  |  |  | (se=0.131) |
|  |  |  | [-0.408 \| 0.106] |
| “web 1st” group # Portugal |  |  | 0.033 |
|  |  |  | (se=0.150) |
|  |  |  | [-0.262 \| 0.328] |
| R^2^ | 0.002^†^ | 0.269*** | 0.270*** |
| corr. R^2^ | 0.001 | 0.264 | 0.264 |
| degrees of freedom (df) | 1 | 10 | 13 |
| Observations | 1,516 | 1,516 | 1,516 |

Displayed are average marginal effects, which are identical to regression coefficients, standard errors and 95% confidence intervals. † p<0.10, * p<0.05, ** p<0.01, *** p<0.001. Data: GGP Pilot Study 2018. Dependent variable: generated indicator, based on the questions “Do you and your partner have children together? Please consider only the children of whom you are a biological mother/ father.”, “How many biological children have you had together?” as well as the according loop questions regarding previous partners. Source: own computation.

**Table 7 Effect of mode on respondents reporting being childless (logit model, average marginal effects)**

|  | Model 1 | Model 2 | Model 3 |
| --- | --- | --- | --- |
| “web 1st” group *(ref.: CAPI)* | 0.050^†^ | 0.039 | 0.061 |
|  | (se=0.028) | (se=0.024) | (se=0.045) |
|  | [-0.005 \| 0.105] | [-0.008 \| 0.085] | [-0.027 \| 0.149] |
| female *(ref.: male)* |  | -0.078*** | -0.046 |
|  |  | (se=0.021) | (se=0.040) |
|  |  | [-0.120 \| -0.036] | [-0.124 \| 0.032] |
| age: 18-30 *(ref.: 31-40)* |  | 0.498*** | 0.499*** |
|  |  | (se=0.025) | (se=0.025) |
|  |  | [0.449 \| 0.547] | [0.450 \| 0.548] |
| age: 41-50 *(ref.: 31-40)* |  | -0.122*** | -0.121*** |
|  |  | (se=0.030) | (se=0.030) |
|  |  | [-0.181 \| -0.063] | [-0.180 \| -0.062] |
| education: low *(ref.: medium)* |  | -0.073* | -0.073* |
|  |  | (se=0.035) | (se=0.035) |
|  |  | [-0.142 \| -0.004] | [-0.142 \| -0.004] |
| education: high *(ref.: medium)* |  | 0.033 | 0.033 |
|  |  | (se=0.023) | (se=0.023) |
|  |  | [-0.012 \| 0.078] | [-0.013 \| 0.078] |
| no national citizenship *(ref.: nat.)* |  | -0.065 | -0.067 |
|  |  | (se=0.050) | (se=0.050) |
|  |  | [-0.163 \| 0.032] | [-0.164 \| 0.031] |
| region: rural *(ref.: urban)* |  | -0.052* | -0.052* |
|  |  | (se=0.022) | (se=0.022) |
|  |  | [-0.095 \| -0.009] | [-0.095 \| -0.009] |
| country: Croatia *(ref.: Germany)* |  | 0.010 | -0.008 |
|  |  | (se=0.026) | (se=0.047) |
|  |  | [-0.040 \| 0.061] | [-0.099 \| 0.083] |
| country: Portugal *(ref.: Germany)* |  | 0.018 | 0.037 |
|  |  | (se=0.031) | (se=0.050) |
|  |  | [-0.043 \| 0.078] | [-0.061 \| 0.134] |
| “web 1st” group # female |  |  | -0.044 |
|  |  |  | (se=0.047) |
|  |  |  | [-0.135 \| 0.048] |
| “web 1st” group # Croatia |  |  | 0.023 |
|  |  |  | (se=0.054) |
|  |  |  | [-0.083 \| 0.130] |
| “web 1st” group # Portugal |  |  | -0.031 |
|  |  |  | (se=0.063) |
|  |  |  | [-0.154 \| 0.092] |
| McFadden R^2^ | 0.002^†^ | 0.267*** | 0.268*** |
| corr. McFadden R^2^ | -0.001^†^ | 0.250*** | 0.248*** |
| Count R^2^ | 0.548^†^ | 0.751*** | 0.749*** |
| corr. Count R^2^ | 0.000^†^ | 0.448*** | 0.445*** |
| degrees of freedom (df) | 1 | 10 | 13 |
| Observations | 1,516 | 1,516 | 1,516 |

Displayed are average marginal effects, standard errors and 95% confidence intervals. † p<0.10, * p<0.05, ** p<0.01, *** p<0.001. Data: GGP Pilot Study 2018. Dependent variable: generated indicator, based on the questions “Do you and your partner have children together? Please consider only the children of whom you are a biological mother/ father.”, “How many biological children have you had together?” as well as the according loop questions regarding previous partners, answer: “0”. Source: own computation.

**Table 8 Effect of mode on reported number of children intended (OLS regression, average marginal effects)**

|  | Model 1 | Model 2 | Model 3 |
| --- | --- | --- | --- |
| “web 1st” group *(ref.: CAPI)* | 0.086 | 0.063 | -0.014 |
|  | (se=0.076) | (se=0.077) | (se=0.141) |
|  | [-0.063 \| 0.235] | [-0.089 \| 0.214] | [-0.291 \| 0.263] |
| female *(ref.: male)* |  | -0.072 | 0.019 |
|  |  | (se=0.071) | (se=0.125) |
|  |  | [-0.212 \| 0.067] | [-0.225 \| 0.264] |
| age: 18-30 *(ref.: 31-40)* |  | 0.098 | 0.107 |
|  |  | (se=0.086) | (se=0.086) |
|  |  | [-0.070 \| 0.266] | [-0.061 \| 0.275] |
| age: 41-50 *(ref.: 31-40)* |  | -0.357*** | -0.351*** |
|  |  | (se=0.088) | (se=0.088) |
|  |  | [-0.530 \| -0.183] | [-0.524 \| -0.178] |
| education: low *(ref.: medium)* |  | -0.054 | -0.045 |
|  |  | (se=0.119) | (se=0.118) |
|  |  | [-0.286 \| 0.179] | [-0.277 \| 0.187] |
| education: high *(ref.: medium)* |  | 0.021 | 0.023 |
|  |  | (se=0.076) | (se=0.077) |
|  |  | [-0.129 \| 0.171] | [-0.127 \| 0.174] |
| no national citizenship *(ref.: nat.)* |  | 0.439** | 0.436** |
|  |  | (se=0.154) | (se=0.154) |
|  |  | [0.136 \| 0.741] | [0.134 \| 0.738] |
| region: rural *(ref.: urban)* |  | 0.088 | 0.081 |
|  |  | (se=0.073) | (se=0.073) |
|  |  | [-0.055 \| 0.231] | [-0.062 \| 0.223] |
| country: Croatia *(ref.: Germany)* |  | 0.112 | 0.066 |
|  |  | (se=0.084) | (se=0.150) |
|  |  | [-0.052 \| 0.276] | [-0.228 \| 0.359] |
| country: Portugal *(ref.: Germany)* |  | -0.144 | -0.453** |
|  |  | (se=0.102) | (se=0.152) |
|  |  | [-0.344 \| 0.055] | [-0.752 \| -0.155] |
| “web 1st” group # female |  |  | -0.133 |
|  |  |  | (se=0.152) |
|  |  |  | [-0.430 \| 0.164] |
| “web 1st” group # Croatia |  |  | 0.092 |
|  |  |  | (se=0.174) |
|  |  |  | [-0.250 \| 0.435] |
| “web 1st” group # Portugal |  |  | 0.551** |
|  |  |  | (se=0.201) |
|  |  |  | [0.156 \| 0.946] |
| R^2^ | 0.001 | 0.045*** | 0.053*** |
| corr. R^2^ | 0.000 | 0.036 | 0.041 |
| degrees of freedom (df) | 1 | 10 | 13 |
| Observations | 1,025 | 1,025 | 1,025 |

Displayed are average marginal effects, which are identical to regression coefficients, standard errors and 95% confidence intervals. † p<0.10, * p<0.05, ** p<0.01, *** p<0.001. Data: GGP Pilot Study 2018. Wording of dependent variable: “How many children – including biological and adoptive children – do you intend to have overall?”. Source: own computation.

**Table 9 Effect of mode on reported number of household members (OLS regression, average marginal effects)**

|  | Model 1 | Model 2 | Model 3 |
| --- | --- | --- | --- |
| “web 1st” group *(ref.: CAPI)* | -0.179* | -0.235** | -0.242 |
|  | (se=0.087) | (se=0.088) | (se=0.167) |
|  | [-0.350 \| -0.007] | [-0.407 \| -0.063] | [-0.570 \| 0.085] |
| female *(ref.: male)* |  | 0.150^†^ | 0.062 |
|  |  | (se=0.079) | (se=0.145) |
|  |  | [-0.004 \| 0.305] | [-0.222 \| 0.345] |
| age: 18-30 *(ref.: 31-40)* |  | -0.494*** | -0.498*** |
|  |  | (se=0.094) | (se=0.095) |
|  |  | [-0.679 \| -0.309] | [-0.683 \| -0.312] |
| age: 41-50 *(ref.: 31-40)* |  | 0.158 | 0.152 |
|  |  | (se=0.097) | (se=0.098) |
|  |  | [-0.034 \| 0.349] | [-0.040 \| 0.343] |
| education: low *(ref.: medium)* |  | 0.010 | 0.018 |
|  |  | (se=0.132) | (se=0.132) |
|  |  | [-0.249 \| 0.269] | [-0.242 \| 0.278] |
| education: high *(ref.: medium)* |  | -0.198* | -0.192* |
|  |  | (se=0.085) | (se=0.085) |
|  |  | [-0.365 \| -0.032] | [-0.359 \| -0.026] |
| no national citizenship *(ref.: nat.)* |  | -0.010 | -0.006 |
|  |  | (se=0.185) | (se=0.185) |
|  |  | [-0.373 \| 0.353] | [-0.369 \| 0.358] |
| region: rural *(ref.: urban)* |  | 0.139^†^ | 0.138^†^ |
|  |  | (se=0.081) | (se=0.081) |
|  |  | [-0.019 \| 0.297] | [-0.020 \| 0.297] |
| country: Croatia *(ref.: Germany)* |  | 0.236* | 0.407* |
|  |  | (se=0.095) | (se=0.172) |
|  |  | [0.050 \| 0.422] | [0.069 \| 0.744] |
| country: Portugal *(ref.: Germany)* |  | -0.300** | -0.359* |
|  |  | (se=0.113) | (se=0.180) |
|  |  | [-0.522 \| -0.077] | [-0.712 \| -0.007] |
| “web 1st” group # female |  |  | 0.123 |
|  |  |  | (se=0.172) |
|  |  |  | [-0.215 \| 0.460] |
| “web 1st” group # Croatia |  |  | -0.224 |
|  |  |  | (se=0.200) |
|  |  |  | [-0.616 \| 0.168] |
| “web 1st” group # Portugal |  |  | 0.102 |
|  |  |  | (se=0.229) |
|  |  |  | [-0.347 \| 0.550] |
| R^2^ | 0.003* | 0.061*** | 0.063*** |
| corr. R^2^ | 0.002 | 0.055 | 0.055 |
| degrees of freedom (df) | 1 | 10 | 13 |
| Observations | 1,525 | 1,525 | 1,525 |

Displayed are average marginal effects, which are identical to regression coefficients, standard errors and 95% confidence intervals. † p<0.10, * p<0.05, ** p<0.01, *** p<0.001. Data: GGP Pilot Study 2018. Dependent variable: generated indicator, based on the questions “Does anybody else live with you [and your partner/children] in this household?”, “How many other people live in your household [excluding you and your partner/children]?” and the information on partner and children in the household. Source: own computation.

**Table 10 Effect of mode on reported number of social network members (OLS regression, average marginal effects)**

|  | Model 1 | Model 2 | Model 3 |
| --- | --- | --- | --- |
| “web 1st” group *(ref.: CAPI)* | -0.271** | -0.337*** | -0.854*** |
|  | (se=0.093) | (se=0.092) | (se=0.174) |
|  | [-0.453 \| -0.089] | [-0.517 \| -0.157] | [-1.195 \| -0.513] |
| female *(ref.: male)* |  | 0.381*** | 0.175 |
|  |  | (se=0.082) | (se=0.151) |
|  |  | [0.219 \| 0.543] | [-0.121 \| 0.470] |
| age: 18-30 *(ref.: 31-40)* |  | -0.051 | -0.056 |
|  |  | (se=0.099) | (se=0.099) |
|  |  | [-0.245 \| 0.142] | [-0.250 \| 0.137] |
| age: 41-50 *(ref.: 31-40)* |  | -0.215* | -0.205* |
|  |  | (se=0.102) | (se=0.102) |
|  |  | [-0.415 \| -0.014] | [-0.404 \| -0.005] |
| education: low *(ref.: medium)* |  | -0.067 | -0.080 |
|  |  | (se=0.138) | (se=0.138) |
|  |  | [-0.338 \| 0.205] | [-0.351 \| 0.191] |
| education: high *(ref.: medium)* |  | 0.586*** | 0.577*** |
|  |  | (se=0.089) | (se=0.088) |
|  |  | [0.412 \| 0.760] | [0.404 \| 0.751] |
| no national citizenship *(ref.: nat.)* |  | -0.513** | -0.495* |
|  |  | (se=0.194) | (se=0.193) |
|  |  | [-0.893 \| -0.133] | [-0.874 \| -0.116] |
| region: rural *(ref.: urban)* |  | -0.148^†^ | -0.157^†^ |
|  |  | (se=0.084) | (se=0.084) |
|  |  | [-0.314 \| 0.018] | [-0.322 \| 0.008] |
| country: Croatia *(ref.: Germany)* |  | -0.330*** | -0.617*** |
|  |  | (se=0.099) | (se=0.179) |
|  |  | [-0.525 \| -0.136] | [-0.969 \| -0.266] |
| country: Portugal *(ref.: Germany)* |  | -0.733*** | -1.226*** |
|  |  | (se=0.119) | (se=0.187) |
|  |  | [-0.966 \| -0.500] | [-1.593 \| -0.859] |
| “web 1st” group # female |  |  | 0.287 |
|  |  |  | (se=0.179) |
|  |  |  | [-0.065 \| 0.639] |
| “web 1st” group # Croatia |  |  | 0.440* |
|  |  |  | (se=0.208) |
|  |  |  | [0.031 \| 0.848] |
| “web 1st” group # Portugal |  |  | 0.806*** |
|  |  |  | (se=0.238) |
|  |  |  | [0.339 \| 1.274] |
| R^2^ | 0.006** | 0.092*** | 0.101*** |
| corr. R^2^ | 0.005 | 0.086 | 0.093 |
| degrees of freedom (df) | 1 | 10 | 13 |
| Observations | 1,525 | 1,525 | 1,525 |

Displayed are average marginal effects, which are identical to regression coefficients, standard errors and 95% confidence intervals. † p<0.10, * p<0.05, ** p<0.01, *** p<0.001. Data: GGP Pilot Study 2018. Wording of dependent variable: “From time to time, most people discuss things that are important to them with others. For example, these may include good or bad things that happen to you, problems you are having, or important concerns you may have. Looking back over the last 12 months, who are the people with whom you typically discuss important personal matters? Please provide the first name and initial of the last name of each of these persons. Please be careful when typing in each name: ensure that you are consistent throughout when entering names”. Source: own computation.

**Table 11 Effect of mode on respondents reporting not having a social network (logit model, average marginal effects)**

|  | Model 1 | Model 2 | Model 3 |
| --- | --- | --- | --- |
| “web 1st” group *(ref.: CAPI)* | 0.174*** | 0.196*** | 0.292*** |
|  | (se=0.024) | (se=0.024) | (se=0.038) |
|  | [0.126 \| 0.222] | [0.149 \| 0.242] | [0.218 \| 0.366] |
| female *(ref.: male)* |  | -0.110*** | -0.042 |
|  |  | (se=0.024) | (se=0.050) |
|  |  | [-0.157 \| -0.064] | [-0.139 \| 0.055] |
| age: 18-30 *(ref.: 31-40)* |  | 0.001 | 0.003 |
|  |  | (se=0.028) | (se=0.028) |
|  |  | [-0.055 \| 0.057] | [-0.053 \| 0.059] |
| age: 41-50 *(ref.: 31-40)* |  | -0.025 | -0.027 |
|  |  | (se=0.029) | (se=0.029) |
|  |  | [-0.082 \| 0.032] | [-0.084 \| 0.030] |
| education: low *(ref.: medium)* |  | 0.012 | 0.017 |
|  |  | (se=0.041) | (se=0.042) |
|  |  | [-0.069 \| 0.093] | [-0.064 \| 0.099] |
| education: high *(ref.: medium)* |  | -0.096*** | -0.093*** |
|  |  | (se=0.025) | (se=0.025) |
|  |  | [-0.145 \| -0.046] | [-0.142 \| -0.043] |
| no national citizenship *(ref.: nat.)* |  | 0.044 | 0.041 |
|  |  | (se=0.058) | (se=0.058) |
|  |  | [-0.069 \| 0.157] | [-0.072 \| 0.155] |
| region: rural *(ref.: urban)* |  | 0.013 | 0.015 |
|  |  | (se=0.024) | (se=0.024) |
|  |  | [-0.035 \| 0.060] | [-0.033 \| 0.062] |
| country: Croatia *(ref.: Germany)* |  | -0.004 | 0.094^†^ |
|  |  | (se=0.028) | (se=0.055) |
|  |  | [-0.059 \| 0.051] | [-0.015 \| 0.202] |
| country: Portugal *(ref.: Germany)* |  | 0.159*** | 0.235*** |
|  |  | (se=0.035) | (se=0.057) |
|  |  | [0.090 \| 0.228] | [0.122 \| 0.347] |
| “web 1st” group # female |  |  | -0.087 |
|  |  |  | (se=0.055) |
|  |  |  | [-0.195 \| 0.022] |
| “web 1st” group # Croatia |  |  | -0.134* |
|  |  |  | (se=0.068) |
|  |  |  | [-0.267 \| -0.001] |
| “web 1st” group # Portugal |  |  | -0.117^†^ |
|  |  |  | (se=0.071) |
|  |  |  | [-0.257 \| 0.022] |
| McFadden R^2^ | 0.023*** | 0.062*** | 0.065*** |
| corr. McFadden R^2^ | 0.020*** | 0.043*** | 0.044*** |
| Count R^2^ | 0.663*** | 0.661*** | 0.659*** |
| corr. Count R^2^ | 0.000*** | -0.006*** | -0.012*** |
| degrees of freedom (df) | 1 | 10 | 13 |
| Observations | 1,525 | 1,525 | 1,525 |

Displayed are average marginal effects, standard errors and 95% confidence intervals. † p<0.10,
* p<0.05, ** p<0.01, *** p<0.001. Data: GGP Pilot Study 2018. Wording of dependent variable: “From time to time, most people discuss things that are important to them with others. For example, these may include good or bad things that happen to you, problems you are having, or important concerns you may have. Looking back over the last 12 months, who are the people with whom you typically discuss important personal matters? Please provide the first name and initial of the last name of each of these persons. Please be careful when typing in each name: ensure that you are consistent throughout when entering names”, no name provided. Source: own computation.

**Table 12 Effect of mode on reported number of previous partners (OLS regression, average marginal effects)**

|  | Model 1 | Model 2 | Model 3 |
| --- | --- | --- | --- |
| “web 1st” group *(ref.: CAPI)* | -0.051 | -0.036 | -0.118^†^ |
|  | (se=0.036) | (se=0.036) | (se=0.068) |
|  | [-0.121 \| 0.020] | [-0.107 \| 0.035] | [-0.251 \| 0.016] |
| female *(ref.: male)* |  | 0.050 | 0.103^†^ |
|  |  | (se=0.033) | (se=0.059) |
|  |  | [-0.014 \| 0.114] | [-0.012 \| 0.218] |
| age: 18-30 *(ref.: 31-40)* |  | -0.152*** | -0.149*** |
|  |  | (se=0.039) | (se=0.039) |
|  |  | [-0.229 \| -0.076] | [-0.225 \| -0.073] |
| age: 41-50 *(ref.: 31-40)* |  | 0.145*** | 0.150*** |
|  |  | (se=0.041) | (se=0.041) |
|  |  | [0.065 \| 0.225] | [0.070 \| 0.230] |
| education: low *(ref.: medium)* |  | 0.073 | 0.069 |
|  |  | (se=0.055) | (se=0.055) |
|  |  | [-0.035 \| 0.181] | [-0.038 \| 0.177] |
| education: high *(ref.: medium)* |  | 0.017 | 0.014 |
|  |  | (se=0.035) | (se=0.035) |
|  |  | [-0.052 \| 0.087] | [-0.055 \| 0.084] |
| no national citizenship *(ref.: nat.)* |  | 0.058 | 0.060 |
|  |  | (se=0.077) | (se=0.076) |
|  |  | [-0.092 \| 0.208] | [-0.090 \| 0.210] |
| region: rural *(ref.: urban)* |  | -0.116*** | -0.118*** |
|  |  | (se=0.034) | (se=0.034) |
|  |  | [-0.182 \| -0.050] | [-0.184 \| -0.052] |
| country: Croatia *(ref.: Germany)* |  | -0.092* | -0.227** |
|  |  | (se=0.039) | (se=0.070) |
|  |  | [-0.168 \| -0.015] | [-0.364 \| -0.090] |
| country: Portugal *(ref.: Germany)* |  | -0.001 | -0.129^†^ |
|  |  | (se=0.048) | (se=0.073) |
|  |  | [-0.094 \| 0.092] | [-0.272 \| 0.014] |
| “web 1st” group # female |  |  | -0.077 |
|  |  |  | (se=0.070) |
|  |  |  | [-0.215 \| 0.061] |
| “web 1st” group # Croatia |  |  | 0.199* |
|  |  |  | (se=0.082) |
|  |  |  | [0.039 \| 0.359] |
| “web 1st” group # Portugal |  |  | 0.215* |
|  |  |  | (se=0.094) |
|  |  |  | [0.029 \| 0.400] |
| R^2^ | 0.001 | 0.054*** | 0.059*** |
| corr. R^2^ | 0.001 | 0.047 | 0.051 |
| degrees of freedom (df) | 1 | 10 | 13 |
| Observations | 1,449 | 1,449 | 1,449 |

Displayed are average marginal effects, which are identical to regression coefficients, standard errors and 95% confidence intervals. † p<0.10, * p<0.05, ** p<0.01, *** p<0.001. Data: GGP Pilot Study 2018. Dependent variable: generated indicator, based on the questions “Besides your current partner or spouse, have you ever before lived together with someone as a couple or have you ever been married?” and “In addition to your current relationship, how many partnerships did you have?”. Source: own computation.

**Table 13 Effect of mode on respondents reporting having no previous partner (logit model, average marginal effects)**

|  | Model 1 | Model 2 | Model 3 |
| --- | --- | --- | --- |
| “web 1st” group *(ref.: CAPI)* | 0.032 | 0.029 | 0.044 |
|  | (se=0.023) | (se=0.023) | (se=0.045) |
|  | [-0.013 \| 0.077] | [-0.015 \| 0.074] | [-0.044 \| 0.132] |
| female *(ref.: male)* |  | -0.049* | -0.083* |
|  |  | (se=0.020) | (se=0.035) |
|  |  | [-0.089 \| -0.010] | [-0.152 \| -0.014] |
| age: 18-30 *(ref.: 31-40)* |  | 0.104*** | 0.103*** |
|  |  | (se=0.023) | (se=0.022) |
|  |  | [0.060 \| 0.149] | [0.059 \| 0.146] |
| age: 41-50 *(ref.: 31-40)* |  | -0.089** | -0.092** |
|  |  | (se=0.028) | (se=0.028) |
|  |  | [-0.144 \| -0.033] | [-0.148 \| -0.037] |
| education: low *(ref.: medium)* |  | -0.019 | -0.016 |
|  |  | (se=0.034) | (se=0.034) |
|  |  | [-0.085 \| 0.048] | [-0.082 \| 0.051] |
| education: high *(ref.: medium)* |  | -0.015 | -0.013 |
|  |  | (se=0.022) | (se=0.022) |
|  |  | [-0.058 \| 0.028] | [-0.056 \| 0.030] |
| no national citizenship *(ref.: nat.)* |  | -0.030 | -0.029 |
|  |  | (se=0.048) | (se=0.048) |
|  |  | [-0.123 \| 0.064] | [-0.123 \| 0.065] |
| region: rural *(ref.: urban)* |  | 0.077*** | 0.077*** |
|  |  | (se=0.021) | (se=0.021) |
|  |  | [0.036 \| 0.117] | [0.037 \| 0.118] |
| country: Croatia *(ref.: Germany)* |  | 0.048^†^ | 0.124** |
|  |  | (se=0.025) | (se=0.046) |
|  |  | [-0.001 \| 0.096] | [0.034 \| 0.213] |
| country: Portugal *(ref.: Germany)* |  | 0.016 | 0.045 |
|  |  | (se=0.031) | (se=0.049) |
|  |  | [-0.044 \| 0.077] | [-0.052 \| 0.142] |
| “web 1st” group # female |  |  | 0.052 |
|  |  |  | (se=0.044) |
|  |  |  | [-0.035 \| 0.139] |
| “web 1st” group # Croatia |  |  | -0.106* |
|  |  |  | (se=0.051) |
|  |  |  | [-0.206 \| -0.005] |
| “web 1st” group # Portugal |  |  | -0.039 |
|  |  |  | (se=0.057) |
|  |  |  | [-0.151 \| 0.074] |
| McFadden R^2^ | 0.001 | 0.061*** | 0.065*** |
| corr. McFadden R^2^ | -0.003 | 0.035*** | 0.035*** |
| Count R^2^ | 0.807 | 0.809*** | 0.810*** |
| corr. Count R^2^ | 0.000 | 0.007*** | 0.011*** |
| degrees of freedom (df) | 1 | 10 | 13 |
| Observations | 1,449 | 1,449 | 1,449 |

Displayed are average marginal effects, standard errors and 95% confidence intervals. † p<0.10,
* p<0.05, ** p<0.01, *** p<0.001. Data: GGP Pilot Study 2018. Dependent variable: generated indicator, based on the questions “Besides your current partner or spouse, have you ever before lived together with someone as a couple or have you ever been married?” and “In addition to your current relationship, how many partnerships did you have?”, answer “0”. Source: own computation.

**Table 14 Effect of mode on respondents reporting having had unprotected sex in the last 12 months (logit model, average marginal effects)**

|  | Model 1 | Model 2 | Model 3 |
| --- | --- | --- | --- |
| “web 1st” group *(ref.: CAPI)* | -0.061* | -0.089** | -0.053 |
|  | (se=0.030) | (se=0.030) | (se=0.057) |
|  | [-0.119 \| -0.002] | [-0.148 \| -0.031] | [-0.164 \| 0.058] |
| female *(ref.: male)* |  | -0.042 | -0.014 |
|  |  | (se=0.027) | (se=0.047) |
|  |  | [-0.095 \| 0.011] | [-0.107 \| 0.079] |
| age: 18-30 *(ref.: 31-40)* |  | -0.197*** | -0.196*** |
|  |  | (se=0.032) | (se=0.032) |
|  |  | [-0.260 \| -0.135] | [-0.259 \| -0.133] |
| age: 41-50 *(ref.: 31-40)* |  | -0.012 | -0.013 |
|  |  | (se=0.035) | (se=0.035) |
|  |  | [-0.081 \| 0.056] | [-0.082 \| 0.055] |
| education: low *(ref.: medium)* |  | -0.027 | -0.024 |
|  |  | (se=0.047) | (se=0.047) |
|  |  | [-0.119 \| 0.065] | [-0.116 \| 0.068] |
| education: high *(ref.: medium)* |  | -0.071* | -0.069* |
|  |  | (se=0.029) | (se=0.029) |
|  |  | [-0.128 \| -0.014] | [-0.126 \| -0.012] |
| no national citizenship *(ref.: nat.)* |  | 0.114^†^ | 0.114^†^ |
|  |  | (se=0.064) | (se=0.064) |
|  |  | [-0.011 \| 0.239] | [-0.011 \| 0.239] |
| region: rural *(ref.: urban)* |  | 0.047^†^ | 0.047^†^ |
|  |  | (se=0.028) | (se=0.028) |
|  |  | [-0.008 \| 0.101] | [-0.008 \| 0.101] |
| country: Croatia *(ref.: Germany)* |  | 0.190*** | 0.219*** |
|  |  | (se=0.032) | (se=0.054) |
|  |  | [0.128 \| 0.252] | [0.113 \| 0.326] |
| country: Portugal *(ref.: Germany)* |  | 0.051 | 0.047 |
|  |  | (se=0.037) | (se=0.054) |
|  |  | [-0.022 \| 0.124] | [-0.059 \| 0.152] |
| “web 1st” group # female |  |  | -0.041 |
|  |  |  | (se=0.058) |
|  |  |  | [-0.154 \| 0.072] |
| “web 1st” group # Croatia |  |  | -0.041 |
|  |  |  | (se=0.068) |
|  |  |  | [-0.174 \| 0.092] |
| “web 1st” group # Portugal |  |  | 0.009 |
|  |  |  | (se=0.077) |
|  |  |  | [-0.141 \| 0.159] |
| McFadden R^2^ | 0.002* | 0.059*** | 0.060*** |
| corr. McFadden R^2^ | -0.001* | 0.038*** | 0.036*** |
| Count R^2^ | 0.585* | 0.628*** | 0.625*** |
| corr. Count R^2^ | 0.000* | 0.103*** | 0.098*** |
| degrees of freedom (df) | 1 | 10 | 13 |
| Observations | 1,257 | 1,257 | 1,257 |

Displayed are average marginal effects, standard errors and 95% confidence intervals. † p<0.10,
* p<0.05, ** p<0.01, *** p<0.001. Data: GGP Pilot Study 2018. Wording of dependent variable:
“Did you regularly have unprotected sexual intercourse over the course of the last 12 months? By ‘regularly’ we mean at least every month”, answer “Yes”. Source: own computation.

**Table 15 Effect of mode on item nonresponse in question on unprotected sex in the last 12 months (logit model, average marginal effects)**

|  | Model 1 | Model 2 | Model 3 |
| --- | --- | --- | --- |
| “web 1st” group *(ref.: CAPI)* | 0.096*** | 0.092*** | 0.095* |
|  | (se=0.019) | (se=0.020) | (se=0.040) |
|  | [0.058 \| 0.133] | [0.054 \| 0.131] | [0.016 \| 0.174] |
| female *(ref.: male)* |  | -0.004 | 0.021 |
|  |  | (se=0.019) | (se=0.043) |
|  |  | [-0.042 \| 0.034] | [-0.063 \| 0.106] |
| age: 18-30 *(ref.: 31-40)* |  | -0.029 | -0.029 |
|  |  | (se=0.023) | (se=0.023) |
|  |  | [-0.074 \| 0.016] | [-0.074 \| 0.016] |
| age: 41-50 *(ref.: 31-40)* |  | 0.011 | 0.009 |
|  |  | (se=0.024) | (se=0.024) |
|  |  | [-0.037 \| 0.059] | [-0.038 \| 0.057] |
| education: low *(ref.: medium)* |  | 0.074^†^ | 0.079* |
|  |  | (se=0.039) | (se=0.039) |
|  |  | [-0.003 \| 0.150] | [0.002 \| 0.156] |
| education: high *(ref.: medium)* |  | -0.057** | -0.055** |
|  |  | (se=0.020) | (se=0.020) |
|  |  | [-0.097 \| -0.018] | [-0.095 \| -0.016] |
| no national citizenship *(ref.: nat.)* |  | 0.052 | 0.055 |
|  |  | (se=0.055) | (se=0.055) |
|  |  | [-0.055 \| 0.160] | [-0.054 \| 0.163] |
| region: rural *(ref.: urban)* |  | 0.005 | 0.004 |
|  |  | (se=0.020) | (se=0.020) |
|  |  | [-0.034 \| 0.044] | [-0.035 \| 0.043] |
| country: Croatia *(ref.: Germany)* |  | 0.092*** | 0.128** |
|  |  | (se=0.022) | (se=0.047) |
|  |  | [0.048 \| 0.136] | [0.036 \| 0.220] |
| country: Portugal *(ref.: Germany)* |  | 0.058* | -0.023 |
|  |  | (se=0.027) | (se=0.044) |
|  |  | [0.005 \| 0.110] | [-0.108 \| 0.063] |
| “web 1st” group # female |  |  | -0.032 |
|  |  |  | (se=0.049) |
|  |  |  | [-0.127 \| 0.063] |
| “web 1st” group # Croatia |  |  | -0.031 |
|  |  |  | (se=0.055) |
|  |  |  | [-0.139 \| 0.077] |
| “web 1st” group # Portugal |  |  | 0.130^†^ |
|  |  |  | (se=0.071) |
|  |  |  | [-0.009 \| 0.270] |
| McFadden R^2^ | 0.015*** | 0.042*** | 0.047*** |
| corr. McFadden R^2^ | 0.011*** | 0.016*** | 0.017*** |
| Count R^2^ | 0.824*** | 0.824*** | 0.824*** |
| corr. Count R^2^ | 0.000*** | 0.000*** | 0.000*** |
| degrees of freedom (df) | 1 | 10 | 13 |
| Observations | 1,525 | 1,525 | 1,525 |

Displayed are average marginal effects, standard errors and 95% confidence intervals. † p<0.10,
* p<0.05, ** p<0.01, *** p<0.001. Data: GGP Pilot Study 2018. Wording of dependent variable:
“Did you regularly have unprotected sexual intercourse over the course of the last 12 months? By ‘regularly’ we mean at least every month”, answer “Yes”. Source: own computation.

**Table 16 Effect of mode on respondents reporting having had sex in the last four weeks (logit model, average marginal effects)**

|  | Model 1 | Model 2 | Model 3 |
| --- | --- | --- | --- |
| “web 1st” group *(ref.: CAPI)* | -0.072** | -0.067* | -0.126* |
|  | (se=0.027) | (se=0.028) | (se=0.049) |
|  | [-0.126 \| -0.019] | [-0.122 \| -0.012] | [-0.223 \| -0.030] |
| female *(ref.: male)* |  | 0.034 | -0.010 |
|  |  | (se=0.026) | (se=0.048) |
|  |  | [-0.018 \| 0.085] | [-0.105 \| 0.085] |
| age: 18-30 *(ref.: 31-40)* |  | -0.162*** | -0.165*** |
|  |  | (se=0.031) | (se=0.032) |
|  |  | [-0.223 \| -0.100] | [-0.227 \| -0.103] |
| age: 41-50 *(ref.: 31-40)* |  | 0.006 | 0.006 |
|  |  | (se=0.031) | (se=0.031) |
|  |  | [-0.054 \| 0.066] | [-0.054 \| 0.066] |
| education: low *(ref.: medium)* |  | -0.065 | -0.069 |
|  |  | (se=0.048) | (se=0.048) |
|  |  | [-0.160 \| 0.029] | [-0.164 \| 0.026] |
| education: high *(ref.: medium)* |  | 0.006 | 0.004 |
|  |  | (se=0.028) | (se=0.028) |
|  |  | [-0.048 \| 0.060] | [-0.050 \| 0.059] |
| no national citizenship *(ref.: nat.)* |  | 0.070 | 0.072 |
|  |  | (se=0.059) | (se=0.059) |
|  |  | [-0.046 \| 0.186] | [-0.044 \| 0.187] |
| region: rural *(ref.: urban)* |  | 0.069* | 0.068* |
|  |  | (se=0.027) | (se=0.027) |
|  |  | [0.016 \| 0.122] | [0.016 \| 0.121] |
| country: Croatia *(ref.: Germany)* |  | -0.018 | -0.055 |
|  |  | (se=0.031) | (se=0.056) |
|  |  | [-0.079 \| 0.043] | [-0.164 \| 0.055] |
| country: Portugal *(ref.: Germany)* |  | -0.015 | -0.047 |
|  |  | (se=0.038) | (se=0.060) |
|  |  | [-0.090 \| 0.060] | [-0.164 \| 0.070] |
| “web 1st” group # female |  |  | 0.061 |
|  |  |  | (se=0.057) |
|  |  |  | [-0.051 \| 0.174] |
| “web 1st” group # Croatia |  |  | 0.052 |
|  |  |  | (se=0.067) |
|  |  |  | [-0.079 \| 0.182] |
| “web 1st” group # Portugal |  |  | 0.052 |
|  |  |  | (se=0.077) |
|  |  |  | [-0.099 \| 0.203] |
| McFadden R^2^ | 0.005** | 0.037*** | 0.039*** |
| corr. McFadden R^2^ | 0.000** | 0.012*** | 0.010*** |
| Count R^2^ | 0.707** | 0.711*** | 0.713*** |
| corr. Count R^2^ | 0.000** | 0.014*** | 0.023*** |
| degrees of freedom (df) | 1 | 10 | 13 |
| Observations | 1,196 | 1,196 | 1,196 |

Displayed are average marginal effects, standard errors and 95% confidence intervals. † p<0.10,
* p<0.05, ** p<0.01, *** p<0.001. Data: GGP Pilot Study 2018. Wording of dependent variable: “When was the last time you had sexual intercourse?”, answers indicating intercourse in the last four weeks. Source: own computation.

**Table 17 Effect of mode on item nonresponse in question on the last time respondent had sex (logit model, average marginal effects)**

|  | Model 1 | Model 2 | Model 3 |
| --- | --- | --- | --- |
| “web 1st” group *(ref.: CAPI)* | 0.083*** | 0.090*** | 0.101* |
|  | (se=0.022) | (se=0.022) | (se=0.042) |
|  | [0.041 \| 0.126] | [0.047 \| 0.133] | [0.018 \| 0.184] |
| female *(ref.: male)* |  | 0.017 | 0.072^†^ |
|  |  | (se=0.021) | (se=0.043) |
|  |  | [-0.024 \| 0.058] | [-0.013 \| 0.157] |
| age: 18-30 *(ref.: 31-40)* |  | -0.032 | -0.030 |
|  |  | (se=0.025) | (se=0.025) |
|  |  | [-0.081 \| 0.017] | [-0.079 \| 0.019] |
| age: 41-50 *(ref.: 31-40)* |  | 0.025 | 0.026 |
|  |  | (se=0.027) | (se=0.027) |
|  |  | [-0.027 \| 0.078] | [-0.026 \| 0.079] |
| education: low *(ref.: medium)* |  | 0.064 | 0.065^†^ |
|  |  | (se=0.039) | (se=0.039) |
|  |  | [-0.013 \| 0.141] | [-0.012 \| 0.142] |
| education: high *(ref.: medium)* |  | -0.035 | -0.035 |
|  |  | (se=0.022) | (se=0.022) |
|  |  | [-0.079 \| 0.009] | [-0.079 \| 0.009] |
| no national citizenship *(ref.: nat.)* |  | 0.084 | 0.084 |
|  |  | (se=0.056) | (se=0.056) |
|  |  | [-0.026 \| 0.194] | [-0.026 \| 0.193] |
| region: rural *(ref.: urban)* |  | -0.011 | -0.011 |
|  |  | (se=0.022) | (se=0.022) |
|  |  | [-0.053 \| 0.031] | [-0.054 \| 0.031] |
| country: Croatia *(ref.: Germany)* |  | 0.024 | -0.001 |
|  |  | (se=0.025) | (se=0.052) |
|  |  | [-0.025 \| 0.073] | [-0.103 \| 0.101] |
| country: Portugal *(ref.: Germany)* |  | 0.050 | 0.004 |
|  |  | (se=0.031) | (se=0.054) |
|  |  | [-0.011 \| 0.111] | [-0.102 \| 0.110] |
| “web 1st” group # female |  |  | -0.073 |
|  |  |  | (se=0.051) |
|  |  |  | [-0.173 \| 0.026] |
| “web 1st” group # Croatia |  |  | 0.036 |
|  |  |  | (se=0.058) |
|  |  |  | [-0.078 \| 0.151] |
| “web 1st” group # Portugal |  |  | 0.066 |
|  |  |  | (se=0.064) |
|  |  |  | [-0.060 \| 0.193] |
| McFadden R^2^ | 0.009*** | 0.021*** | 0.023*** |
| corr. McFadden R^2^ | 0.005*** | -0.002*** | -0.004*** |
| Count R^2^ | 0.784*** | 0.784*** | 0.784*** |
| corr. Count R^2^ | 0.000*** | 0.000*** | 0.000*** |
| degrees of freedom (df) | 1 | 10 | 13 |
| Observations | 1,525 | 1,525 | 1,525 |

Displayed are average marginal effects, standard errors and 95% confidence intervals. † p<0.10,
* p<0.05, ** p<0.01, *** p<0.001. Data: GGP Pilot Study 2018. Wording of dependent variable: “When was the last time you had sexual intercourse?”. Source: own computation.

**Table 18 Effect of mode on respondents reporting assuming to be infertile (logit model, average marginal effects)**

|  | Model 1 | Model 2 | Model 3 |
| --- | --- | --- | --- |
| “web 1st” group *(ref.: CAPI)* | -0.028 | -0.017 | -0.056 |
|  | (se=0.021) | (se=0.019) | (se=0.044) |
|  | [-0.069 \| 0.012] | [-0.056 \| 0.021] | [-0.142 \| 0.031] |
| female *(ref.: male)* |  | 0.060*** | 0.026 |
|  |  | (se=0.017) | (se=0.029) |
|  |  | [0.026 \| 0.094] | [-0.030 \| 0.082] |
| age: 18-30 *(ref.: 31-40)* |  | -0.027 | -0.028^†^ |
|  |  | (se=0.016) | (se=0.016) |
|  |  | [-0.058 \| 0.005] | [-0.060 \| 0.004] |
| age: 41-50 *(ref.: 31-40)* |  | 0.151*** | 0.150*** |
|  |  | (se=0.024) | (se=0.024) |
|  |  | [0.103 \| 0.198] | [0.102 \| 0.198] |
| education: low *(ref.: medium)* |  | 0.065^†^ | 0.065^†^ |
|  |  | (se=0.034) | (se=0.034) |
|  |  | [-0.002 \| 0.133] | [-0.002 \| 0.133] |
| education: high *(ref.: medium)* |  | -0.011 | -0.010 |
|  |  | (se=0.019) | (se=0.019) |
|  |  | [-0.047 \| 0.026] | [-0.047 \| 0.026] |
| no national citizenship *(ref.: nat.)* |  | 0.006 | 0.008 |
|  |  | (se=0.044) | (se=0.044) |
|  |  | [-0.080 \| 0.091] | [-0.079 \| 0.095] |
| region: rural *(ref.: urban)* |  | -0.006 | -0.006 |
|  |  | (se=0.018) | (se=0.018) |
|  |  | [-0.042 \| 0.030] | [-0.043 \| 0.030] |
| country: Croatia *(ref.: Germany)* |  | -0.002 | 0.007 |
|  |  | (se=0.021) | (se=0.034) |
|  |  | [-0.043 \| 0.038] | [-0.060 \| 0.074] |
| country: Portugal *(ref.: Germany)* |  | 0.021 | 0.014 |
|  |  | (se=0.027) | (se=0.038) |
|  |  | [-0.031 \| 0.073] | [-0.060 \| 0.088] |
| “web 1st” group # female |  |  | 0.057 |
|  |  |  | (se=0.039) |
|  |  |  | [-0.018 \| 0.133] |
| “web 1st” group # Croatia |  |  | -0.014 |
|  |  |  | (se=0.042) |
|  |  |  | [-0.097 \| 0.069] |
| “web 1st” group # Portugal |  |  | 0.011 |
|  |  |  | (se=0.049) |
|  |  |  | [-0.085 \| 0.107] |
| McFadden R^2^ | 0.003 | 0.120*** | 0.124*** |
| corr. McFadden R^2^ | -0.005 | 0.074*** | 0.069*** |
| Count R^2^ | 0.892 | 0.892*** | 0.892*** |
| corr. Count R^2^ | 0.000 | 0.000*** | 0.000*** |
| degrees of freedom (df) | 1 | 10 | 13 |
| Observations | 1,128 | 1,128 | 1,128 |

Displayed are average marginal effects, standard errors and 95% confidence intervals. † p<0.10,
* p<0.05, ** p<0.01, *** p<0.001. Data: GGP Pilot Study 2018. Wording of dependent variable: “Some people are not physically able to have children. As far as you know, is it physically possible for you, yourself, to have a/another baby? ... definitely not, probably not, probably yes, definitely yes”, combination of answers “Definitely not” and “Probably not”. Source: own computation.

**Table 19 Effect of mode on item nonresponse in question on subjective infertility (logit model, average marginal effects)**

|  | Model 1 | Model 2 | Model 3 |
| --- | --- | --- | --- |
| “web 1st” group *(ref.: CAPI)* | 0.084*** | 0.080*** | 0.062 |
|  | (se=0.023) | (se=0.024) | (se=0.049) |
|  | [0.038 \| 0.130] | [0.033 \| 0.127] | [-0.035 \| 0.158] |
| female *(ref.: male)* |  | -0.007 | 0.042 |
|  |  | (se=0.022) | (se=0.044) |
|  |  | [-0.051 \| 0.037] | [-0.044 \| 0.128] |
| age: 18-30 *(ref.: 31-40)* |  | 0.091*** | 0.093*** |
|  |  | (se=0.027) | (se=0.027) |
|  |  | [0.038 \| 0.145] | [0.040 \| 0.147] |
| age: 41-50 *(ref.: 31-40)* |  | -0.024 | -0.022 |
|  |  | (se=0.026) | (se=0.026) |
|  |  | [-0.075 \| 0.028] | [-0.074 \| 0.029] |
| education: low *(ref.: medium)* |  | 0.061 | 0.061 |
|  |  | (se=0.041) | (se=0.041) |
|  |  | [-0.019 \| 0.142] | [-0.020 \| 0.141] |
| education: high *(ref.: medium)* |  | -0.051* | -0.051* |
|  |  | (se=0.024) | (se=0.024) |
|  |  | [-0.097 \| -0.004] | [-0.097 \| -0.005] |
| no national citizenship *(ref.: nat.)* |  | 0.132* | 0.132* |
|  |  | (se=0.060) | (se=0.060) |
|  |  | [0.014 \| 0.251] | [0.015 \| 0.250] |
| region: rural *(ref.: urban)* |  | -0.008 | -0.009 |
|  |  | (se=0.023) | (se=0.023) |
|  |  | [-0.053 \| 0.037] | [-0.054 \| 0.036] |
| country: Croatia *(ref.: Germany)* |  | 0.117*** | 0.066 |
|  |  | (se=0.026) | (se=0.051) |
|  |  | [0.066 \| 0.168] | [-0.034 \| 0.167] |
| country: Portugal *(ref.: Germany)* |  | 0.109*** | 0.050 |
|  |  | (se=0.032) | (se=0.054) |
|  |  | [0.046 \| 0.171] | [-0.055 \| 0.155] |
| “web 1st” group # female |  |  | -0.066 |
|  |  |  | (se=0.051) |
|  |  |  | [-0.167 \| 0.035] |
| “web 1st” group # Croatia |  |  | 0.078 |
|  |  |  | (se=0.061) |
|  |  |  | [-0.041 \| 0.197] |
| “web 1st” group # Portugal |  |  | 0.096 |
|  |  |  | (se=0.068) |
|  |  |  | [-0.036 \| 0.229] |
| McFadden R^2^ | 0.007*** | 0.036*** | 0.038*** |
| corr. McFadden R^2^ | 0.003*** | 0.016*** | 0.014*** |
| Count R^2^ | 0.740*** | 0.741*** | 0.741*** |
| corr. Count R^2^ | 0.000*** | 0.005*** | 0.005*** |
| degrees of freedom (df) | 1 | 10 | 13 |
| Observations | 1,525 | 1,525 | 1,525 |

Displayed are average marginal effects, standard errors and 95% confidence intervals. † p<0.10,
* p<0.05, ** p<0.01, *** p<0.001. Data: GGP Pilot Study 2018. Wording of dependent variable: “Some people are not physically able to have children. As far as you know, is it physically possible for you, yourself, to have a/another baby? ... definitely not, probably not, probably yes, definitely yes”, combination of answers “Definitely not” and “Probably not”. Source: own computation.

**Table 20 Effect of mode on respondents reporting using contraceptives (logit model, average marginal effects)**

|  | Model 1 | Model 2 | Model 3 |
| --- | --- | --- | --- |
| “web 1st” group *(ref.: CAPI)* | -0.020 | -0.031 | 0.004 |
|  | (se=0.030) | (se=0.029) | (se=0.051) |
|  | [-0.079 \| 0.039] | [-0.088 \| 0.026] | [-0.096 \| 0.105] |
| female *(ref.: male)* |  | 0.314*** | 0.282*** |
|  |  | (se=0.026) | (se=0.049) |
|  |  | [0.263 \| 0.365] | [0.185 \| 0.379] |
| age: 18-30 *(ref.: 31-40)* |  | -0.045 | -0.047 |
|  |  | (se=0.031) | (se=0.031) |
|  |  | [-0.106 \| 0.016] | [-0.108 \| 0.014] |
| age: 41-50 *(ref.: 31-40)* |  | 0.076* | 0.074* |
|  |  | (se=0.032) | (se=0.032) |
|  |  | [0.013 \| 0.139] | [0.011 \| 0.137] |
| education: low *(ref.: medium)* |  | -0.052 | -0.051 |
|  |  | (se=0.044) | (se=0.044) |
|  |  | [-0.140 \| 0.035] | [-0.138 \| 0.036] |
| education: high *(ref.: medium)* |  | 0.004 | 0.004 |
|  |  | (se=0.028) | (se=0.028) |
|  |  | [-0.051 \| 0.059] | [-0.051 \| 0.059] |
| no national citizenship *(ref.: nat.)* |  | -0.201*** | -0.201*** |
|  |  | (se=0.059) | (se=0.059) |
|  |  | [-0.318 \| -0.085] | [-0.317 \| -0.085] |
| region: rural *(ref.: urban)* |  | 0.010 | 0.011 |
|  |  | (se=0.027) | (se=0.027) |
|  |  | [-0.042 \| 0.063] | [-0.041 \| 0.064] |
| country: Croatia *(ref.: Germany)* |  | -0.081** | -0.034 |
|  |  | (se=0.031) | (se=0.058) |
|  |  | [-0.141 \| -0.021] | [-0.147 \| 0.079] |
| country: Portugal *(ref.: Germany)* |  | -0.167*** | -0.091 |
|  |  | (se=0.037) | (se=0.059) |
|  |  | [-0.239 \| -0.096] | [-0.206 \| 0.023] |
| “web 1st” group # female |  |  | 0.045 |
|  |  |  | (se=0.057) |
|  |  |  | [-0.066 \| 0.156] |
| “web 1st” group # Croatia |  |  | -0.073 |
|  |  |  | (se=0.067) |
|  |  |  | [-0.204 \| 0.058] |
| “web 1st” group # Portugal |  |  | -0.125^†^ |
|  |  |  | (se=0.074) |
|  |  |  | [-0.269 \| 0.020] |
| McFadden R^2^ | 0.000 | 0.100*** | 0.102*** |
| corr. McFadden R^2^ | -0.003 | 0.079*** | 0.078*** |
| Count R^2^ | 0.578 | 0.679*** | 0.684*** |
| corr. Count R^2^ | 0.000 | 0.239*** | 0.250*** |
| degrees of freedom (df) | 1 | 10 | 13 |
| Observations | 1,286 | 1,286 | 1,286 |

Displayed are average marginal effects, standard errors and 95% confidence intervals. † p<0.10,
* p<0.05, ** p<0.01, *** p<0.001. Data: GGP Pilot Study 2018. Wording of dependent variable: “Are you or your partner/spouse using or doing any of these things to prevent pregnancy at this time? Please name all of the things you use or do. ... Condom, Pills, intra uterine device (coil, loop), diaphragm/ cervical cap, foam/ cream/ jelly/ suppository, injectables (e.g. Depo Provera), implants (e.g. Norplant), Persona, hormonal emergency contraception afterwards (morning after pill), withdrawal, safe period method (rhythm)”, any one option mentioned. Source: own computation.

**Table 21 Effect of mode on respondents reporting that men are doing an equal share of laundry (logit model, average marginal effects)**

|  | Model 1 | Model 2 | Model 3 |
| --- | --- | --- | --- |
| “web 1st” group *(ref.: CAPI)* | -0.060^†^ | -0.054^†^ | -0.058 |
|  | (se=0.031) | (se=0.031) | (se=0.057) |
|  | [-0.120 \| 0.001] | [-0.115 \| 0.006] | [-0.170 \| 0.054] |
| female *(ref.: male)* |  | -0.042 | -0.008 |
|  |  | (se=0.028) | (se=0.047) |
|  |  | [-0.097 \| 0.012] | [-0.099 \| 0.083] |
| age: 18-30 *(ref.: 31-40)* |  | 0.085* | 0.086* |
|  |  | (se=0.042) | (se=0.042) |
|  |  | [0.002 \| 0.168] | [0.004 \| 0.168] |
| age: 41-50 *(ref.: 31-40)* |  | -0.006 | -0.002 |
|  |  | (se=0.028) | (se=0.029) |
|  |  | [-0.062 \| 0.050] | [-0.058 \| 0.054] |
| education: low *(ref.: medium)* |  | -0.050 | -0.051 |
|  |  | (se=0.038) | (se=0.038) |
|  |  | [-0.125 \| 0.025] | [-0.126 \| 0.024] |
| education: high *(ref.: medium)* |  | 0.055^†^ | 0.056^†^ |
|  |  | (se=0.030) | (se=0.030) |
|  |  | [-0.003 \| 0.114] | [-0.003 \| 0.115] |
| no national citizenship *(ref.: nat.)* |  | 0.058 | 0.055 |
|  |  | (se=0.063) | (se=0.063) |
|  |  | [-0.066 \| 0.181] | [-0.068 \| 0.178] |
| region: rural *(ref.: urban)* |  | -0.077** | -0.078** |
|  |  | (se=0.028) | (se=0.028) |
|  |  | [-0.131 \| -0.023] | [-0.132 \| -0.024] |
| country: Croatia *(ref.: Germany)* |  | -0.047 | -0.086 |
|  |  | (se=0.030) | (se=0.055) |
|  |  | [-0.107 \| 0.012] | [-0.194 \| 0.022] |
| country: Portugal *(ref.: Germany)* |  | 0.094* | 0.052 |
|  |  | (se=0.047) | (se=0.072) |
|  |  | [0.002 \| 0.186] | [-0.088 \| 0.192] |
| “web 1st” group # female |  |  | -0.051 |
|  |  |  | (se=0.057) |
|  |  |  | [-0.163 \| 0.060] |
| “web 1st” group # Croatia |  |  | 0.059 |
|  |  |  | (se=0.067) |
|  |  |  | [-0.072 \| 0.190] |
| “web 1st” group # Portugal |  |  | 0.064 |
|  |  |  | (se=0.074) |
|  |  |  | [-0.081 \| 0.208] |
| McFadden R^2^ | 0.005* | 0.051*** | 0.053*** |
| corr. McFadden R^2^ | -0.003* | 0.005*** | -0.001*** |
| Count R^2^ | 0.813* | 0.812*** | 0.811*** |
| corr. Count R^2^ | 0.000* | -0.007*** | -0.013*** |
| degrees of freedom (df) | 1 | 10 | 13 |
| Observations | 814 | 814 | 814 |

Displayed are average marginal effects, standard errors and 95% confidence intervals. † p<0.10,
* p<0.05, ** p<0.01, *** p<0.001. Data: GGP Pilot Study 2018. Wording of dependent variable: “Please indicate who does the following tasks in your household ... doing the laundry ... always me, usually me, equally me and partner, usually partner, always partner, always or usually someone else”, combination of the answers “Equally me and partner” plus the two options with the male partner doing more, the answer “Always or usually someone else” is excluded from the analyses. Source: own computation.

**Table 22 Effect of mode on respondents reporting that men are doing an equal share of preparing daily meals (logit model, average marginal effects)**

|  | Model 1 | Model 2 | Model 3 |
| --- | --- | --- | --- |
| “web 1st” group *(ref.: CAPI)* | -0.023 | -0.027 | -0.061 |
|  | (se=0.036) | (se=0.036) | (se=0.068) |
|  | [-0.094 \| 0.048] | [-0.098 \| 0.044] | [-0.194 \| 0.071] |
| female *(ref.: male)* |  | -0.086* | -0.087 |
|  |  | (se=0.034) | (se=0.060) |
|  |  | [-0.152 \| -0.020] | [-0.205 \| 0.030] |
| age: 18-30 *(ref.: 31-40)* |  | 0.133** | 0.133** |
|  |  | (se=0.049) | (se=0.049) |
|  |  | [0.036 \| 0.230] | [0.036 \| 0.229] |
| age: 41-50 *(ref.: 31-40)* |  | -0.028 | -0.026 |
|  |  | (se=0.035) | (se=0.035) |
|  |  | [-0.097 \| 0.041] | [-0.096 \| 0.043] |
| education: low *(ref.: medium)* |  | -0.092^†^ | -0.093^†^ |
|  |  | (se=0.052) | (se=0.052) |
|  |  | [-0.194 \| 0.009] | [-0.195 \| 0.009] |
| education: high *(ref.: medium)* |  | 0.015 | 0.013 |
|  |  | (se=0.036) | (se=0.036) |
|  |  | [-0.055 \| 0.085] | [-0.057 \| 0.084] |
| no national citizenship *(ref.: nat.)* |  | -0.127* | -0.127* |
|  |  | (se=0.064) | (se=0.065) |
|  |  | [-0.253 \| -0.001] | [-0.253 \| -0.000] |
| region: rural *(ref.: urban)* |  | -0.122*** | -0.123*** |
|  |  | (se=0.034) | (se=0.034) |
|  |  | [-0.188 \| -0.056] | [-0.189 \| -0.057] |
| country: Croatia *(ref.: Germany)* |  | -0.054 | -0.100 |
|  |  | (se=0.038) | (se=0.068) |
|  |  | [-0.127 \| 0.020] | [-0.234 \| 0.035] |
| country: Portugal *(ref.: Germany)* |  | 0.088^†^ | 0.057 |
|  |  | (se=0.050) | (se=0.080) |
|  |  | [-0.011 \| 0.187] | [-0.098 \| 0.213] |
| “web 1st” group # female |  |  | 0.002 |
|  |  |  | (se=0.071) |
|  |  |  | [-0.137 \| 0.141] |
| “web 1st” group # Croatia |  |  | 0.066 |
|  |  |  | (se=0.082) |
|  |  |  | [-0.094 \| 0.227] |
| “web 1st” group # Portugal |  |  | 0.048 |
|  |  |  | (se=0.094) |
|  |  |  | [-0.137 \| 0.233] |
| McFadden R^2^ | 0.000 | 0.045*** | 0.045*** |
| corr. McFadden R^2^ | -0.005 | 0.010*** | 0.005*** |
| Count R^2^ | 0.657 | 0.670*** | 0.673*** |
| corr. Count R^2^ | 0.000 | 0.036*** | 0.047*** |
| degrees of freedom (df) | 1 | 10 | 13 |
| Observations | 811 | 811 | 811 |

Displayed are average marginal effects, standard errors and 95% confidence intervals. † p<0.10,
* p<0.05, ** p<0.01, *** p<0.001. Data: GGP Pilot Study 2018. Wording of dependent variable: “Please indicate who does the following tasks in your household ... preparing daily meals ... always me, usually me, equally me and partner, usually partner, always partner, always or usually someone else”, combination of the answers “Equally me and partner” plus the two options with the male partner doing more, the answer “Always or usually someone else” is excluded from the analyses. Source: own computation.

**Table 23 Effect of mode on reported life satisfaction (OLS regression, average marginal effects)**

|  | Model 1 | Model 2 | Model 3 |
| --- | --- | --- | --- |
| “web 1st” group *(ref.: CAPI)* | -0.584*** | -0.569*** | -0.534** |
|  | (se=0.099) | (se=0.102) | (se=0.190) |
|  | [-0.779 \| -0.389] | [-0.770 \| -0.369] | [-0.907 \| -0.160] |
| female *(ref.: male)* |  | 0.111 | 0.018 |
|  |  | (se=0.093) | (se=0.166) |
|  |  | [-0.071 \| 0.294] | [-0.307 \| 0.343] |
| age: 18-30 *(ref.: 31-40)* |  | -0.091 | -0.095 |
|  |  | (se=0.112) | (se=0.112) |
|  |  | [-0.310 \| 0.129] | [-0.315 \| 0.125] |
| age: 41-50 *(ref.: 31-40)* |  | -0.202^†^ | -0.204^†^ |
|  |  | (se=0.116) | (se=0.116) |
|  |  | [-0.430 \| 0.026] | [-0.432 \| 0.024] |
| education: low *(ref.: medium)* |  | -0.163 | -0.167 |
|  |  | (se=0.159) | (se=0.159) |
|  |  | [-0.474 \| 0.148] | [-0.479 \| 0.145] |
| education: high *(ref.: medium)* |  | 0.144 | 0.142 |
|  |  | (se=0.100) | (se=0.100) |
|  |  | [-0.053 \| 0.340] | [-0.055 \| 0.339] |
| no national citizenship *(ref.: nat.)* |  | 0.215 | 0.214 |
|  |  | (se=0.217) | (se=0.218) |
|  |  | [-0.212 \| 0.641] | [-0.213 \| 0.640] |
| region: rural *(ref.: urban)* |  | 0.198* | 0.200* |
|  |  | (se=0.095) | (se=0.095) |
|  |  | [0.011 \| 0.386] | [0.013 \| 0.387] |
| country: Croatia *(ref.: Germany)* |  | -0.067 | -0.031 |
|  |  | (se=0.110) | (se=0.198) |
|  |  | [-0.283 \| 0.150] | [-0.419 \| 0.357] |
| country: Portugal *(ref.: Germany)* |  | -0.048 | 0.175 |
|  |  | (se=0.136) | (se=0.206) |
|  |  | [-0.314 \| 0.218] | [-0.230 \| 0.579] |
| “web 1st” group # female |  |  | 0.139 |
|  |  |  | (se=0.200) |
|  |  |  | [-0.253 \| 0.532] |
| “web 1st” group # Croatia |  |  | -0.071 |
|  |  |  | (se=0.231) |
|  |  |  | [-0.524 \| 0.382] |
| “web 1st” group # Portugal |  |  | -0.392 |
|  |  |  | (se=0.270) |
|  |  |  | [-0.921 \| 0.137] |
| R^2^ | 0.025*** | 0.035*** | 0.037*** |
| corr. R^2^ | 0.024 | 0.028 | 0.027 |
| degrees of freedom (df) | 1 | 10 | 13 |
| Observations | 1,376 | 1,376 | 1,376 |

Displayed are average marginal effects, which are identical to regression coefficients, standard errors and 95% confidence intervals. † p<0.10, * p<0.05, ** p<0.01, *** p<0.001. Data: GGP Pilot Study 2018. Wording of dependent variable: “All things considered, how satisfied are you with your life as a whole nowadays? ... from 0 (‘extremely dissatisfied’) to 10 (‘extremely satisfied’)”. Source: own computation.

**Table 24 Effect of mode on reported happiness (OLS regression, average marginal effects)**

|  | Model 1 | Model 2 | Model 3 |
| --- | --- | --- | --- |
| “web 1st” group *(ref.: CAPI)* | -0.550*** | -0.569*** | -0.469** |
|  | (se=0.089) | (se=0.092) | (se=0.172) |
|  | [-0.726 \| -0.375] | [-0.749 \| -0.388] | [-0.806 \| -0.131] |
| female *(ref.: male)* |  | 0.153^†^ | 0.108 |
|  |  | (se=0.084) | (se=0.149) |
|  |  | [-0.012 \| 0.318] | [-0.184 \| 0.400] |
| age: 18-30 *(ref.: 31-40)* |  | -0.166 | -0.167^†^ |
|  |  | (se=0.101) | (se=0.101) |
|  |  | [-0.364 \| 0.033] | [-0.366 \| 0.031] |
| age: 41-50 *(ref.: 31-40)* |  | -0.157 | -0.162 |
|  |  | (se=0.105) | (se=0.105) |
|  |  | [-0.363 \| 0.049] | [-0.368 \| 0.044] |
| education: low *(ref.: medium)* |  | -0.021 | -0.019 |
|  |  | (se=0.144) | (se=0.145) |
|  |  | [-0.304 \| 0.262] | [-0.302 \| 0.265] |
| education: high *(ref.: medium)* |  | 0.165^†^ | 0.169^†^ |
|  |  | (se=0.090) | (se=0.091) |
|  |  | [-0.012 \| 0.342] | [-0.009 \| 0.346] |
| no national citizenship *(ref.: nat.)* |  | 0.081 | 0.080 |
|  |  | (se=0.199) | (se=0.199) |
|  |  | [-0.309 \| 0.471] | [-0.310 \| 0.470] |
| region: rural *(ref.: urban)* |  | 0.106 | 0.109 |
|  |  | (se=0.086) | (se=0.086) |
|  |  | [-0.063 \| 0.275] | [-0.060 \| 0.278] |
| country: Croatia *(ref.: Germany)* |  | 0.019 | 0.135 |
|  |  | (se=0.100) | (se=0.178) |
|  |  | [-0.177 \| 0.214] | [-0.213 \| 0.484] |
| country: Portugal *(ref.: Germany)* |  | -0.119 | 0.067 |
|  |  | (se=0.122) | (se=0.186) |
|  |  | [-0.359 \| 0.121] | [-0.298 \| 0.431] |
| “web 1st” group # female |  |  | 0.069 |
|  |  |  | (se=0.180) |
|  |  |  | [-0.285 \| 0.423] |
| “web 1st” group # Croatia |  |  | -0.180 |
|  |  |  | (se=0.208) |
|  |  |  | [-0.588 \| 0.229] |
| “web 1st” group # Portugal |  |  | -0.322 |
|  |  |  | (se=0.243) |
|  |  |  | [-0.799 \| 0.156] |
| R^2^ | 0.027*** | 0.036*** | 0.038*** |
| corr. R^2^ | 0.026 | 0.029 | 0.029 |
| degrees of freedom (df) | 1 | 10 | 13 |
| Observations | 1,376 | 1,376 | 1,376 |

Displayed are average marginal effects, which are identical to regression coefficients, standard errors and 95% confidence intervals. † p<0.10, * p<0.05, ** p<0.01, *** p<0.001. Data: GGP Pilot Study 2018. Wording of dependent variable: “Taking all things together, how happy would you say you are? ... from 0 (‘extremely unhappy’) to 10 (‘extremely happy’)”. Source: own computation.

**Table 25 Mode differences for key demographic indicators (numbers to the graphs displayed in Figure 3)**

|  | | value | 95% confidence interval, | |
| --- | --- | --- | --- | --- |
|  | | (ratio or mean) | lower end | upper end |
| **(1) share of respondents reporting having a partner** | | | | |
| Germany | *reference group (CAPI)* | .760 | .695 | .816 |
|  | *experimental group (web first)* | .759 | .708 | .803 |
|  | *CAWI cases within exp. group* | .776 | .720 | .823 |
| Croatia | *reference group (CAPI)* | .762 | .686 | .825 |
|  | *experimental group (web first)* | .741 | .705 | .775 |
|  | *CAWI cases within exp. group* | .745 | .708 | .779 |
| Portugal | *reference group (CAPI)* | .579 | .489 | .663 |
|  | *experimental group (web first)* | .684 | .615 | .746 |
|  | *CAWI cases within exp. group* | .684 | .615 | .746 |
| **(2) share of respondents reporting having a same sex relationship** | | | | |
| Germany | *reference group (CAPI)* | .005 | .001 | .036 |
|  | *experimental group (web first)* | .019 | .009 | .042 |
|  | *CAWI cases within exp. group* | .024 | .011 | .052 |
| Croatia | *reference group (CAPI)* | .000 | - | - |
|  | *experimental group (web first)* | .003 | .001 | .013 |
|  | *CAWI cases within exp. group* | .003 | .001 | .014 |
| Portugal | *reference group (CAPI)* | .017 | .004 | .065 |
|  | *experimental group (web first)* | .011 | .003 | .042 |
|  | *CAWI cases within exp. group* | .011 | .003 | .042 |
| **(3) share of respondents reporting cohabiting with a partner** | | | | |
| Germany | *reference group (CAPI)* | .661 | .592 | .725 |
|  | *experimental group (web first)* | .641 | .586 | .692 |
|  | *CAWI cases within exp. group* | .671 | .610 | .726 |
| Croatia | *reference group (CAPI)* | .594 | .512 | .672 |
|  | *experimental group (web first)* | .577 | .537 | .616 |
|  | *CAWI cases within exp. group* | .577 | .536 | .617 |
| Portugal | *reference group (CAPI)* | .492 | .403 | .581 |
|  | *experimental group (web first)* | .561 | .490 | .631 |
|  | *CAWI cases within exp. group* | .561 | .490 | .631 |
| **(4) share of respondents reporting being married** | | | | |
| Germany | *reference group (CAPI)* | .474 | .404 | .545 |
|  | *experimental group (web first)* | .521 | .465 | .577 |
|  | *CAWI cases within exp. group* | .544 | .482 | .605 |
| Croatia | *reference group (CAPI)* | .552 | .470 | .632 |
|  | *experimental group (web first)* | .466 | .426 | .507 |
|  | *CAWI cases within exp. group* | .468 | .427 | .509 |
| Portugal | *reference group (CAPI)* | .342 | .262 | .431 |
|  | *experimental group (web first)* | .353 | .288 | .424 |
|  | *CAWI cases within exp. group* | .353 | .288 | .424 |
| **(5) average reported number of biological children** | | | | |
| Germany | *reference group (CAPI)* | .953 | .790 | 1.117 |
|  | *experimental group (web first)* | .949 | .814 | 1.083 |
|  | *CAWI cases within exp. group* | .965 | .813 | 1.117 |
| Croatia | *reference group (CAPI)* | 1.067 | .864 | 1.270 |
|  | *experimental group (web first)* | .797 | .711 | .882 |
|  | *CAWI cases within exp. group* | .785 | .700 | .871 |
| Portugal | *reference group (CAPI)* | .810 | .606 | 1.014 |
|  | *experimental group (web first)* | .781 | .626 | .935 |
|  | *CAWI cases within exp. group* | .781 | .626 | .935 |
| **(6) share of respondents reporting being childless** | | | | |
| Germany | *reference group (CAPI)* | .518 | .448 | .588 |
|  | *experimental group (web first)* | .537 | .481 | .591 |
|  | *CAWI cases within exp. group* | .535 | .474 | .595 |
| Croatia | *reference group (CAPI)* | .497 | .417 | .576 |
|  | *experimental group (web first)* | .589 | .550 | .626 |
|  | *CAWI cases within exp. group* | .591 | .552 | .629 |
| Portugal | *reference group (CAPI)* | .545 | .456 | .632 |
|  | *experimental group (web first)* | .545 | .474 | .615 |
|  | *CAWI cases within exp. group* | .545 | .474 | .616 |
| **(7) average reported number of children intended** | | | | |
| Germany | *reference group (CAPI)* | 2.097 | 1.935 | 2.260 |
|  | *experimental group (web first)* | 2.008 | 1.871 | 2.145 |
|  | *CAWI cases within exp. group* | 1.995 | 1.835 | 2.155 |
| Croatia | *reference group (CAPI)* | 2.082 | 1.834 | 2.331 |
|  | *experimental group (web first)* | 2.081 | 1.965 | 2.196 |
|  | *CAWI cases within exp. group* | 2.063 | 1.948 | 2.178 |
| Portugal | *reference group (CAPI)* | 1.644 | 1.394 | 1.895 |
|  | *experimental group (web first)* | 2.056 | 1.819 | 2.293 |
|  | *CAWI cases within exp. group* | 2.056 | 1.819 | 2.293 |
| **(8) average reported number of household members** | | | | |
| Germany | *reference group (CAPI)* | 3.161 | 2.972 | 3.349 |
|  | *experimental group (web first)* | 2.956 | 2.801 | 3.111 |
|  | *CAWI cases within exp. group* | 2.912 | 2.740 | 3.083 |
| Croatia | *reference group (CAPI)* | 3.517 | 3.227 | 3.807 |
|  | *experimental group (web first)* | 3.053 | 2.922 | 3.183 |
|  | *CAWI cases within exp. group* | 3.037 | 2.904 | 3.169 |
| Portugal | *reference group (CAPI)* | 2.662 | 2.419 | 2.904 |
|  | *experimental group (web first)* | 2.670 | 2.472 | 2.868 |
|  | *CAWI cases within exp. group* | 2.670 | 2.472 | 2.868 |
| **(9) average reported number of social network members** | | | | |
| Germany | *reference group (CAPI)* | 2.280 | 2.033 | 2.527 |
|  | *experimental group (web first)* | 1.569 | 1.374 | 1.764 |
|  | *CAWI cases within exp. group* | 1.506 | 1.286 | 1.726 |
| Croatia | *reference group (CAPI)* | 1.664 | 1.431 | 1.897 |
|  | *experimental group (web first)* | 1.439 | 1.315 | 1.562 |
|  | *CAWI cases within exp. group* | 1.422 | 1.296 | 1.548 |
| Portugal | *reference group (CAPI)* | .917 | .752 | 1.082 |
|  | *experimental group (web first)* | 1.099 | .865 | 1.332 |
|  | *CAWI cases within exp. group* | 1.099 | .865 | 1.332 |
| **(10) share of respondents reporting not having a social network** | | | | |
| Germany | *reference group (CAPI)* | .145 | .102 | .202 |
|  | *experimental group (web first)* | .406 | .353 | .461 |
|  | *CAWI cases within exp. group* | .452 | .393 | .513 |
| Croatia | *reference group (CAPI)* | .219 | .159 | .294 |
|  | *experimental group (web first)* | .376 | .340 | .415 |
|  | *CAWI cases within exp. group* | .382 | .345 | .421 |
| Portugal | *reference group (CAPI)* | .406 | .326 | .491 |
|  | *experimental group (web first)* | .557 | .488 | .624 |
|  | *CAWI cases within exp. group* | .557 | .488 | .624 |
| **(11) average reported number of previous partners** | | | | |
| Germany | *reference group (CAPI)* | .401 | .287 | .515 |
|  | *experimental group (web first)* | .256 | .190 | .321 |
|  | *CAWI cases within exp. group* | .256 | .181 | .331 |
| Croatia | *reference group (CAPI)* | .199 | .117 | .281 |
|  | *experimental group (web first)* | .218 | .173 | .262 |
|  | *CAWI cases within exp. group* | .218 | .174 | .262 |
| Portugal | *reference group (CAPI)* | .267 | .166 | .367 |
|  | *experimental group (web first)* | .315 | .195 | .436 |
|  | *CAWI cases within exp. group* | .315 | .195 | .436 |
| **(12) share of respondents reporting having no previous partner** | | | | |
| Germany | *reference group (CAPI)* | .740 | .673 | .797 |
|  | *experimental group (web first)* | .810 | .762 | .850 |
|  | *CAWI cases within exp. group* | .820 | .767 | .863 |
| Croatia | *reference group (CAPI)* | .844 | .774 | .895 |
|  | *experimental group (web first)* | .829 | .796 | .858 |
|  | *CAWI cases within exp. group* | .825 | .791 | .854 |
| Portugal | *reference group (CAPI)* | .783 | .701 | .848 |
|  | *experimental group (web first)* | .821 | .756 | .872 |
|  | *CAWI cases within exp. group* | .821 | .756 | .872 |
| **(13) share of respondents reporting having had unprotected sex in the last 12 months** | | | | |
| Germany | *reference group (CAPI)* | .367 | .299 | .441 |
|  | *experimental group (web first)* | .311 | .259 | .369 |
|  | *CAWI cases within exp. group* | .300 | .242 | .364 |
| Croatia | *reference group (CAPI)* | .605 | .515 | .689 |
|  | *experimental group (web first)* | .467 | .423 | .512 |
|  | *CAWI cases within exp. group* | .463 | .418 | .509 |
| Portugal | *reference group (CAPI)* | .429 | .340 | .522 |
|  | *experimental group (web first)* | .375 | .300 | .457 |
|  | *CAWI cases within exp. group* | .375 | .300 | .457 |
| **(14) share of item-nonresponse in question on unprotected sex in the last 12 months** | | | | |
| Germany | *reference group (CAPI)* | .083 | .051 | .131 |
|  | *experimental group (web first)* | .151 | .116 | .195 |
|  | *CAWI cases within exp. group* | .169 | .128 | .219 |
| Croatia | *reference group (CAPI)* | .201 | .144 | .273 |
|  | *experimental group (web first)* | .262 | .230 | .298 |
|  | *CAWI cases within exp. group* | .263 | .230 | .299 |
| Portugal | *reference group (CAPI)* | .158 | .105 | .230 |
|  | *experimental group (web first)* | .291 | .232 | .357 |
|  | *CAWI cases within exp. group* | .291 | .232 | .357 |
| **(15) share of respondents reporting having had sex in the last four weeks** | | | | |
| Germany | *reference group (CAPI)* | .772 | .701 | .830 |
|  | *experimental group (web first)* | .691 | .630 | .746 |
|  | *CAWI cases within exp. group* | .697 | .629 | .756 |
| Croatia | *reference group (CAPI)* | .738 | .653 | .808 |
|  | *experimental group (web first)* | .677 | .634 | .718 |
|  | *CAWI cases within exp. group* | .678 | .634 | .720 |
| Portugal | *reference group (CAPI)* | .747 | .653 | .823 |
|  | *experimental group (web first)* | .707 | .624 | .778 |
|  | *CAWI cases within exp. group* | .707 | .624 | .778 |
| **(16) share of item-nonresponse in question on the last time you had sex** | | | | |
| Germany | *reference group (CAPI)* | .161 | .115 | .219 |
|  | *experimental group (web first)* | .226 | .184 | .276 |
|  | *CAWI cases within exp. group* | .230 | .183 | .285 |
| Croatia | *reference group (CAPI)* | .181 | .127 | .251 |
|  | *experimental group (web first)* | .278 | .245 | .314 |
|  | *CAWI cases within exp. group* | .277 | .243 | .314 |
| Portugal | *reference group (CAPI)* | .256 | .189 | .337 |
|  | *experimental group (web first)* | .345 | .283 | .413 |
|  | *CAWI cases within exp. group* | .345 | .283 | .413 |
| **(17) share of respondents reporting assuming to be infertile** | | | | |
| Germany | *reference group (CAPI)* | .115 | .073 | .175 |
|  | *experimental group (web first)* | .107 | .074 | .152 |
|  | *CAWI cases within exp. group* | .102 | .068 | .152 |
| Croatia | *reference group (CAPI)* | .142 | .089 | .219 |
|  | *experimental group (web first)* | .088 | .064 | .119 |
|  | *CAWI cases within exp. group* | .091 | .067 | .123 |
| Portugal | *reference group (CAPI)* | .129 | .075 | .214 |
|  | *experimental group (web first)* | .122 | .075 | .193 |
|  | *CAWI cases within exp. group* | .122 | .075 | .193 |
| **(18) share of item-nonresponse in question on subjective infertility** | | | | |
| Germany | *reference group (CAPI)* | .187 | .138 | .248 |
|  | *experimental group (web first)* | .208 | .166 | .256 |
|  | *CAWI cases within exp. group* | .215 | .169 | .269 |
| Croatia | *reference group (CAPI)* | .242 | .180 | .317 |
|  | *experimental group (web first)* | .345 | .309 | .382 |
|  | *CAWI cases within exp. group* | .349 | .313 | .388 |
| Portugal | *reference group (CAPI)* | .301 | .229 | .384 |
|  | *experimental group (web first)* | .394 | .329 | .463 |
|  | *CAWI cases within exp. group* | .394 | .329 | .463 |
| **(19) share of respondents reporting using contraceptives** | | | | |
| Germany | *reference group (CAPI)* | .607 | .532 | .677 |
|  | *experimental group (web first)* | .635 | .577 | .689 |
|  | *CAWI cases within exp. group* | .632 | .568 | .692 |
| Croatia | *reference group (CAPI)* | .605 | .513 | .691 |
|  | *experimental group (web first)* | .547 | .504 | .590 |
|  | *CAWI cases within exp. group* | .551 | .507 | .594 |
| Portugal | *reference group (CAPI)* | .474 | .384 | .565 |
|  | *experimental group (web first)* | .429 | .358 | .503 |
|  | *CAWI cases within exp. group* | .429 | .358 | .503 |
| **(20) share of respondents reporting that men are doing an equal share of laundry** | | | | |
| Germany | *reference group (CAPI)* | .252 | .183 | .336 |
|  | *experimental group (web first)* | .173 | .127 | .233 |
|  | *CAWI cases within exp. group* | .179 | .128 | .244 |
| Croatia | *reference group (CAPI)* | .148 | .086 | .243 |
|  | *experimental group (web first)* | .145 | .110 | .188 |
|  | *CAWI cases within exp. group* | .146 | .111 | .190 |
| Portugal | *reference group (CAPI)* | .286 | .177 | .427 |
|  | *experimental group (web first)* | .275 | .188 | .383 |
|  | *CAWI cases within exp. group* | .275 | .188 | .383 |
| **(21) share of people reporting that men are doing an equal share of preparing daily meals** | | | | |
| Germany | *reference group (CAPI)* | .376 | .295 | .464 |
|  | *experimental group (web first)* | .318 | .256 | .387 |
|  | *CAWI cases within exp. group* | .337 | .269 | .413 |
| Croatia | *reference group (CAPI)* | .280 | .190 | .392 |
|  | *experimental group (web first)* | .312 | .262 | .366 |
|  | *CAWI cases within exp. group* | .308 | .258 | .362 |
| Portugal | *reference group (CAPI)* | .426 | .302 | .560 |
|  | *experimental group (web first)* | .402 | .305 | .508 |
|  | *CAWI cases within exp. group* | .402 | .305 | .508 |
| **(22) average reported life satisfaction (on a scale from 0 to 10)** | | | | |
| Germany | *reference group (CAPI)* | 8.232 | 8.035 | 8.430 |
|  | *experimental group (web first)* | 7.732 | 7.552 | 7.911 |
|  | *CAWI cases within exp. group* | 7.723 | 7.533 | 7.912 |
| Croatia | *reference group (CAPI)* | 8.148 | 7.854 | 8.442 |
|  | *experimental group (web first)* | 7.630 | 7.459 | 7.802 |
|  | *CAWI cases within exp. group* | 7.625 | 7.454 | 7.796 |
| Portugal | *reference group (CAPI)* | 8.353 | 8.146 | 8.560 |
|  | *experimental group (web first)* | 7.548 | 7.276 | 7.820 |
|  | *CAWI cases within exp. group* | 7.548 | 7.275 | 7.820 |
| **(23) average reported happiness (on a scale from 0 to 10)** | | | | |
| Germany | *reference group (CAPI)* | 8.229 | 8.048 | 8.411 |
|  | *experimental group (web first)* | 7.768 | 7.613 | 7.923 |
|  | *CAWI cases within exp. group* | 7.747 | 7.583 | 7.911 |
| Croatia | *reference group (CAPI)* | 8.312 | 8.092 | 8.532 |
|  | *experimental group (web first)* | 7.724 | 7.566 | 7.883 |
|  | *CAWI cases within exp. group* | 7.700 | 7.538 | 7.862 |
| Portugal | *reference group (CAPI)* | 8.259 | 8.074 | 8.443 |
|  | *experimental group (web first)* | 7.544 | 7.278 | 7.810 |
|  | *CAWI cases within exp. group* | 7.544 | 7.278 | 7.810 |

Indicators: (1) “Do you have a partner at the moment, that is to say someone with whom you have had a relationship for at least 3 months?”, answer “Yes”, (2) Generated indicator, based on the questions “Sex: Male, Female, Other” and “Is your partner ... Male, Female, Other”. There are no combinations of “Other”/”Other” in the data. Missing answers in the survey question were replaced by population register information, if available. (3) “Does your partner live with you in the same household?”, answer “Yes”, (4) Generated indicator, based on the questions “Are you and your partner legally married?” and “Are you and your partner living in a registered partnership?”, answer “Yes”. (5/6) Generated indicator, based on the questions “Do you and your partner have children together? Please consider only the children of whom you are a biological mother/ father.”, “How many biological children have you had together?” as well as the according loop questions regarding previous partners. (7) “How many children – including biological and adoptive children – do you intend to have overall?”, (8) Generated indicator, based on the questions “Does anybody else live with you [and your partner/children] in this household?”, “How many other people live in your household [excluding you and your partner/children]?” and the information on partner and children in the household. (9/10) “From time to time, most people discuss things that are important to them with others. For example, these may include good or bad things that happen to you, problems you are having, or important concerns you may have. Looking back over the last 12 months, who are the people with whom you typically discuss important personal matters? Please provide the first name and initial of the last name of each of these persons. Please be careful when typing in each name: ensure that you are consistent throughout when entering names”. (11/12) Generated indicator, based on the questions “Besides your current partner or spouse, have you ever before lived together with someone as a couple or have you ever been married?” and “In addition to your current relationship, how many partnerships did you have?”. (13/14) “Did you regularly have unprotected sexual intercourse over the course of the last 12 months? By 'regularly' we mean at least every month”, answer “Yes”. (15/16) “When was the last time you had sexual intercourse?”, answers indicating intercourse in the last four weeks. (17/18) “Some people are not physically able to have children. As far as you know, is it physically possible for you, yourself, to have a/another baby? ... definitely not, probably not, probably yes, definitely yes”, combination of answers “Definitely not” and “Probably not”. (19) “Are you or your partner/spouse using or doing any of these things to prevent pregnancy at this time? Please name all of the things you use or do. ... Condom, Pills, intra uterine device (coil, loop), diaphragm/ cervical cap, foam/ cream/ jelly/ suppository, injectables (e.g. Depo Provera), implants (e.g. Norplant), Persona, hormonal emergency contraception afterwards (morning after pill), withdrawal, safe period method (rhythm)” – any one option mentioned. (20) “Please indicate who does the following tasks in your household ... doing the laundry ... always me, usually me, equally me and partner, usually partner, always partner, always or usually someone else”, combination of the answers “Equally me and partner” plus the two options with the male partner doing more, the answer “Always or usually someone else” is excluded from the analyses, (21) “... preparing daily meals ...”. (22) “All things considered, how satisfied are you with your life as a whole nowadays? ... from 0 ('extremely dissatisfied') to 10 ('extremely satisfied')”, (23) “Taking all things together, how happy would you say you are? ... from 0 ('extremely unhappy') to 10 ('extremely happy’)”. Data: GGP pilot-study 2018. Unweighted data. Own calculations.
